# A systematic review of multimodal brain age studies: Uncovering a divergence between model accuracy and utility

**DOI:** 10.1016/j.patter.2023.100712

**Published:** 2023-04-14

**Authors:** Robert J. Jirsaraie, Aaron J. Gorelik, Martins M. Gatavins, Denis A. Engemann, Ryan Bogdan, Deanna M. Barch, Aristeidis Sotiras

**Affiliations:** 1 Division of Computational and Data Sciences, Washington University in St. Louis, St. Louis, MO, USA; 2 Department of Psychological & Brain Sciences, Washington University in St. Louis, St. Louis, MO, USA; 3 Undergraduate Neuroscience Program, School of Arts & Sciences, University of Pennsylvania, Philadelphia, PA, USA; 4 Roche Pharma Research and Early Development, Neuroscience and Rare Diseases, Roche Innovation Center Basel, F. Hoffmann-La Roche, Ltd., Basel, Switzerland; 5 Université Paris-Saclay, Inria, CEA, Palaiseau, France; 6 Department of Radiology and Institute for Informatics, Washington University School of Medicine in St. Louis, St. Louis, MO, USA

**Keywords:** brain age, multimodal imaging, machine learning, systematic review

## Abstract

Brain aging is a complex, multifaceted process that can be challenging to model in ways that are accurate and clinically useful. One of the most common approaches has been to apply machine learning to neuroimaging data with the goal of predicting age in a data-driven manner. Building on initial brain age studies that were derived solely from T1-weighted scans (i.e., unimodal), recent studies have incorporated features across multiple imaging modalities (i.e., “multimodal”). In this systematic review, we show that unimodal and multimodal models have distinct advantages. Multimodal models are the most accurate and sensitive to differences in chronic brain disorders. In contrast, unimodal models from functional magnetic resonance imaging were most sensitive to differences across a broad array of phenotypes. Altogether, multimodal imaging has provided us valuable insight for improving the accuracy of brain age models, but there is still much untapped potential with regard to achieving widespread clinical utility.

## The bigger picture

Establishing standardized biomarkers of brain aging would have immense utility for quantifying the risks of adverse life outcomes, which may lead to more precise and personalized interventions. An increasingly common approach for deriving such biomarkers is to train machine-learning models on a diverse set of neuroimaging features with the goal of accurately estimating a person’s biological age. Further, the difference between biological and chronological ages has been shown to be a useful metric that is sensitive to many phenotypes. However, no definitive guidelines exist to help researchers decide which set of neural properties would improve model accuracy and utility. Accordingly, we conducted a systematic review of multimodal brain age studies to identify which neuroimaging features provided the largest added value. We found that the multimodal models had an increased capacity to predict age, which did not translate to widespread improvement in clinical utility.

## Teaser

In theory, providing brain age models with complimentary information about neural properties and brain organization should result in more nuanced predictions that can better reflect individual differences. In practice, we found that the primary advantage of multimodal models was an increased capacity to predict age, but their brain age gaps did not exhibit widespread improvements in detecting individual differences across a broad array of phenotypes.

## Introduction

Advances in computational resources and machine-learning architecture have empowered researchers to investigate brain aging in a data-driven manner.^1^ It has become increasingly clear that applying machine learning to neuroimaging data can yield accurate estimates about the biological age of a person (i.e., brain age), which can serve as a biomarker for evaluating brain health.^2^ Many studies have demonstrated that discrepancies between brain and chronological ages (i.e., brain age gaps) can be sensitive to differences across a variety of phenotypes, including cognitive functioning,^3^ physical health,^4^ mental disorders,^5^ and biomedical conditions.^2^ As such, brain age gaps can be useful for delineating many brain-behavior relationships, which we operationalize as “model utility.” Given the broad implications of brain age models, it is essential to find ways of improving their clinical applicability such that they can be applied to assess the risks of atypical neurodevelopment and age-related neurodegeneration. One promising avenue for improving brain age models has been to combine features from multiple modalities and across distinct types of neural properties. By generating brain age models with complimentary information about how the brain alters over time (e.g., brain activation and connectivity), their predictions may capture more variation between individuals.^6^ Motivated by the potential advantages that could be obtained from multimodal imaging, we conducted the first systematic review of multimodal brain age studies with a specific focus on evaluating their accuracy and utility.

The first multimodal brain age study was published on December 25, 2011, and incorporated data from three types of magnetic resonance imaging (MRI) sequences: T1-weighted, T2-weighted, and diffusion-weighting scans. Brown et al.^7^ found that the neural features extracted from each of these modalities exhibited unique developmental trajectories throughout adolescences. More importantly, there was an alignment between the years when a given set of features exhibited the most age-related differences and the time when those features contributed the most to the predictive power of their multimodal brain age model. Further, their multimodal model explained more than 92% of the variance in age and yielded mean absolute errors that were as low as 1.03 years. The accuracy of this model underscored the potential advantages of using multimodal data. Since this initial report, there have been over 200 brain age-related articles that utilized or referenced the term “multimodal,” a number that is increasing at an accelerated rate each year (Figure S1). It is expected that this trend will continue, as recent data-sharing requirements for NIH-funded projects^8^ and open science initiatives^9^ will provide easier access to large multimodal imaging datasets. Additionally, continual advancements in harmonization tools may enable researchers to aggregate even larger samples for training and validating brain age models.^10^ These precedents demonstrate the relevance for a review about the potential benefits of using multimodal imaging, especially in the context of brain age modeling.

The brain age framework is an actively evolving field with a wide array of methods to select from for training and validating models. Specifically, there are numerous options to decide between the type of predictive algorithm, validation technique, imaging modality, preprocessing pipeline, spatial templates/atlases, and specific neuroimaging features to extract. Thus far, there are resources to help researchers in selecting predictive algorithms^11^^,^^12^^,^^13^ and validation techniques^14^^,^^15^ but no definitive guidelines regarding which neuroimaging modalities and neural properties to use. This is concerning because there is a vast number of neuroimaging-derived measurements available for both structural and functional data (e.g., cortical thickness, functional connectivity, mean diffusivity, etc.), thereby making it difficult to choose the best set of features to achieve a particular goal. Additionally, recent brain age studies have demonstrated that the most accurate models tend to not be the most useful at detecting differences in cognitive functioning.^16^^,^^17^ Therefore, it is not only essential for researchers to be deliberate when selecting neuroimaging properties but also when selecting which performance metrics to use for evaluating their brain age model(s). To the best of our knowledge, there are no extensive guidelines to assist researchers in navigating these methodological decisions in a principled manner. Although this systematic review is unlikely to completely fill this gap in the literature, we believe that examining the potential benefits of multimodal brain age models would be a conducive starting point.

Through this systematic review, we investigated whether multimodal brain age models had improved accuracy and utility relative to models that were derived from a single imaging source (i.e., unimodal). We explored the following aims across all relevant studies using a quantitative synthesis approach.^18^ We first evaluated which imaging modalities derived the most accurate brain age models (e.g., lowest prediction errors between chronological and brain ages) and whether model accuracy varied with the number of input features (aim 1). Qualitative assessments were conducted to further understand which specific feature types and regions of interest most contributed to the predictive power of brain age models. Additionally, we identified several brain age models whose predictions did not significantly vary with age, which is a well-known issue referred to as prediction bias.^19^ Secondly, we repeated the quantitative synthesis procedure to investigate model utility (aim 2). Subsequently, we qualitatively evaluated whether the utility of brain age models depends on the phenotype in question as well as identified which phenotypes were most strongly correlated with brain age gaps. Lastly, we illustrated the relationship between model accuracy and utility both within each study and across this body of literature (aim 3). Altogether, this review examined which specific neuroimaging features provided the largest added value for both model accuracy and utility. Such insight may prove useful for understanding how best to model age-related pathology using a brain age framework.

## Methods

**Figure 1:**
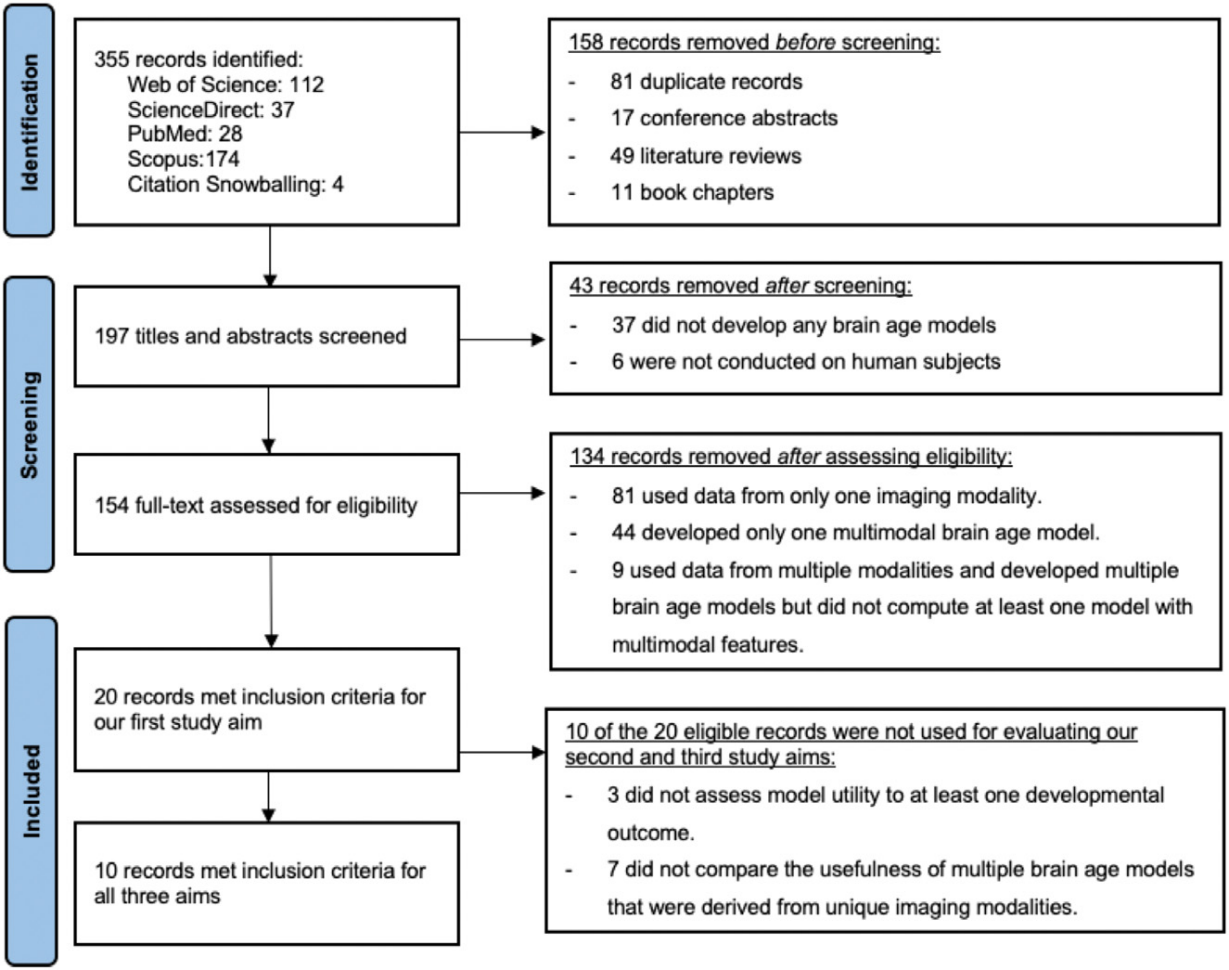
Flow diagram depicting the identification, screening, and eligibility of the **relevant** studies for this systematic review Our literature searches uncovered 197 unique empirical records that were screened. Briefly, studies were excluded if they did not compare multiple brain age models using multiple imaging sources. A total of twenty studies met the inclusion criteria for our first study aim, and of these, ten met our inclusion criteria for all three aims.

This systematic review was conducted in accordance with the Preferred Reporting Items for Systematic Reviews and Meta-analyses (PRISMA)^20^ whenever possible. As such, our four-step PRISMA flow diagram is displayed in Figure 1.

### Information sources and search strategy

On May 16, 2022, literature searches were conducted across four databases (Web of Science, ScienceDirect, PubMed, and Scopus) without any limits to publication date.

We aggregated a total of 351 reports that used the term “brain age” in their title, abstract, or keywords and included the term “multimodal” anywhere throughout the article, including the references. The references for these articles were also screened yielding four additional relevant reports. After removing duplicates (n = 81) and non-empirical investigations (n = 77), we obtained 197 studies whose titles and abstracts were screened by one co-author (M.M.G.). Studies were excluded if they did not train a statistical model to predict age from neuroimaging data (n = 37) or were not conducted on human subjects (n = 6). This screening process yielded 154 studies whose full text was independently assessed for eligibility by two authors (M.M.G. and R.J.J.). Any discrepancies (n = 1) were discussed and resolved through consensus.

### Eligibility criteria

Given our interest in assessing whether multimodal features improved the accuracy and utility of brain age models, we selected studies that developed multiple models across a variety of modalities, with each model being derived from a distinct set of neuroimaging features. Studies comparing the accuracy and utility of unimodal vs. multimodal brain age models were particularly relevant for this review. Therefore, we excluded all studies that only used data from a single imaging modality (n = 81), developed only one multimodal brain age model and did not compare with any unimodal models (n = 44), or did not include at least one multimodal brain age model in their comparison of model accuracy (n = 9). Ultimately, twenty studies met our inclusion criteria, but only a subset of these studies evaluated both the accuracy and utility of their models. Specifically, ten of these studies either did not evaluate individual differences in any phenotype (n = 3) or only assessed the utility of their multimodal model without comparing its performance with their unimodal models (n = 7). Thus, our assessment of model utility was based on a subset of ten studies, and our review of model accuracy utilized all twenty studies.

### Data extraction

Information from each study was compiled and organized into three categories. First, descriptive information regarding the study design and sample characteristics was gathered (Table 1). These data included the sample size, age distribution, phenotype(s), data source(s), validation technique(s), and predictive algorithm(s). Second, the neuroimaging modalities and specific feature types were collected (Table S1). This body of literature encompassed data across multiple MRI sequences, positron emission tomography (PET), magnetoencephalography (MEG), and electroencephalography (EEG). Lastly, the relevant meta-data from each brain age model across all studies were extracted to conduct a quantitative synthesis.^18^ For each brain age model, the following information was recorded: neuroimaging modality; feature type; number of input features; mean absolute error (i.e., operationalized as model accuracy); Pearson correlation between chronological and brain ages; coefficient of determination between chronological and brain ages; phenotype (i.e., cognition, psychopathology, etc.); correlation between a phenotype of interest and brain age gaps (i.e., operationalized as model utility); and reported effect size metrics (i.e., Pearson correlation, Cohen’s D, etc.). This aggregated set of information is accessible via the following GitHub repository: https://zenodo.org/badge/latestdoi/549260376.^21^^,^^22^

**Table 1:** Study design and sample characteristics

Study	Sample size	Age range (years)	Data source	Phenotype	Validation technique	Predictive algorithm
Erus et al.^23^	621	8–22	PNC	cognition	10-fold	SVR
Liem et al.^24^	2,354 + 475	18–85	LIFE, NKI	cognition	5-fold	SVR + multisource RF
Richard et al.^25^	612 + 265	18–88	CamCAN, StrokeMRI	cognition	10-fold	GTB
Cole^2^	17,461	45–80	UK Biobank	biomedical, cognition, lifestyle	10-fold	LASSO
de Lange et al.^26^	610 + 27,157	60–85	Whitehall II, UK Biobank	biomedical	repeated 10-fold	GTB
Engemann et al.^27^	674	18–87	CamCAN	anxiety, biomedical, cognition, depression	10-fold	stacked ensemble ML (ridge + RF)
Galdi et al. (2020)^28^	105	0.44–0.81	local	preterm/term birth	repeated 5-fold	ENET
Hu et al. (2020)^29^	178	0.08–2.33	UNC/UMN Baby Connectome Project	–	nested CV	NN
Niu et al.^12^	839	8–21	PNC	anxiety and PTSD, cognition	nested CV	ridge, SVR, GPR, DNN
Zhang et al. (2020)^30^	1,493	3–21	PING	cognition	10-fold	non-projective dictionary learning
Dadi et al.^31^	11,175	40–70	UK Biobank	cognition, neuroticism (only for one model)	nested CV	RF
Dunas et al.^32^	351	25–85	Betula longitudinal study	cognition, education, fitness	10-fold	BRR, LASSO, ENET, SVR, RVR, GPR
Luna et al. (2021)^33^	489	5–17	HBN	cognition, general psychopathology	5-fold	stacked ensemble ML (GTB, GLM, RF, MLP, SEML)
Rokicki et al.^34^	1,244	18–86	TOP, NorCog, StrokeMRI	Alzheimer’s disease, bipolar disorder, cognitive impairment, schizophrenia	10-fold	RF
Xifra-Porxas et al.^35^	613	18–88	CamCAN	–	nested CV	RF
Chen et al.^36^	829	14–92	local community	schizophrenia	10-fold	feedforward cascade NN, GPR
Chen et al.^37^	636 + 482	18–88	CamCAN, local community	biomedical, cognition	10-fold	previously trained feedforward cascade NN
Huang et al. (2022)^38^	343	20–60	local community	schizophrenia	leave one out	LASSO MLR
Ramduny et al.^39^	50	65–84	local community	lifestyle (sleep)	repeated 5-fold	multiple regression
Yu et al.^40^	196 + 91	60–85	ADNI, local community	cognitive impairment, genetics	repeated 5-fold	ridge

PTSD, post-traumatic stress disorder; CV, cross-validation. Predictive algorithms: SVR, support vector regression; RF, random forest regression; ENET, elastic net regression; GTB, gradient tree boosting regression; LASSO, least absolute shrinkage and selection operator regression; NN, neural network; GPR, Gaussian process regression, DNN, deep neural network; NPDL, non-projective dictionary learning; BRR, Bayesian ridge regression; RVR, relevance vector regression; GLM, general linear model; MLP, multilayer perceptron; SEML, stacked ensemble machine learning; OLS, ordinary least squares regression; MLR, multilinear regression. Data source: PNC, Philadelphia Neurodevelopmental Cohort; PING, Pediatric Imaging, Neurocognition, and Genetics Data Repository; LIFE, Leipzig Research Center for Civilization Diseases Adult Study; NKI, Nathan Kline Institute – Rockland Sample; CamCAN, Cambridge Center for Aging and Neuroscience; TOP, Thematically Organized Psychosis study; NorCog, Norwegian Registry for Persons Assessed for Cognitive Symptoms; ADNI, Alzheimer’s Disease Neuroimaging Initiative; HBN, Healthy Brain Network.

### Data synthesis

As discussed in prior reviews of the brain age framework,^41^ several confounds need to be accounted for when interpreting differences between studies. It has been demonstrated that the accuracy of brain age models varies as a function of sample size,^42^ age distribution,^41^ developmental stage,^43^ predictive algorithm,^13^ and correction methods to remove prediction biases.^44^ This heterogeneity can obscure our ability to infer how the predictive power of a brain age model is related to certain imaging modalities and their corresponding neural properties. Additionally, brain age models have been trained at various scales of neuroimaging data, including voxel-level data from spatial maps,^11^ extracted properties from regional atlases,^45^ and higher-order covariance components derived via factor analysis methods.^31^ To ensure that our results were not driven by these unwanted sources of variation, we minimum/maximum (min/max) scaled the mean absolute errors and the number of input features across all models within a given study. Additionally, we reverse scored the scaled mean absolute errors to make our results easier to interpret (i.e., being in the same direction as model utility). Therefore, the brain age models with a score of 1 were the most accurate within a given study, while those with a 0 were the least accurate. We based our assessment of model accuracy on the mean absolute errors as opposed to the coefficient of determination for two reasons: (1) it has been demonstrated that correlations are artificially weaker when the age range of the training and testing samples is restricted,^14^^,^^15^ and (2) correlations between chronological and brain ages were only reported for twelve of the twenty included studies (Table S2).

### Effect measures

The studies included in this review evaluated the utility of their models across a wide range of phenotypes (e.g., cognitive functioning, mental disorder), consisting of dimensional and categorical measures. Consequently, different types of effect sizes were reported, including h-statistics,^24^ Pearson correlations,^25^^,^^32^^,^^39^^,^^40^ Cohen’s D,^34^^,^^36^ and standardized beta coefficients.^27^^,^^37^^,^^41^ We standardized these indices by converting all measures of effect size to Pearson correlations and subsequently taking their absolute values. These values were used to quantify a model’s utility or more specifically their capacity to detect differences for a given phenotype, regardless of the direction of the association (Table S3). If such information could not be extracted from a research article, it was computed from publicly available data^25^^,^^31^ or the corresponding author was contacted via e-mail.^27^^,^^40^ As a result, the relevant data were obtained across all twenty studies, thereby bypassing issues of missingness.

### Statistical analyses

R v.4.0.2.^46^ was used for analytical and visual purposes. Specifically, we used software from the following packages: *effectsize*,^47^ *ggplot2*,^48^ *lmer*,^49^ and *lmerTest*.^50^ All code and data pertaining to this review are available through the following GitHub repository: https://zenodo.org/badge/latestdoi/549260376.

Our first set of analyses were conducted across twenty brain age studies and investigated differences in the accuracy of brain age models. As such, we used mixed-effects analysis of variance to test whether model accuracy differed between imaging modalities: anova(lmer(accuracy∼modality+(1|study))). Additionally, we used a mixed-effects linear model to test whether model accuracy varied as a function of the number of neuroimaging features used to train the models: lmer(accuracy∼dimensionality+(1|study)). Sensitivity analyses were conducted to further evaluate whether the potential association between model accuracy and dimensionality was moderated by sample size, lmer(accuracy∼dimensionality∗samplesize+(1|study)), or imaging modality, lmer(accuracy∼dimensionality∗modality+(1|study)). For each mixed-effects model outlined above, we used a random intercept that grouped observations by the study they were obtained from.

A second set of analyses were conducted across a subset of ten studies and investigated differences in the utility of brain age models. The same analytical procedure that was used to investigate model accuracy was also used to investigate model utility, except we used a random intercept that grouped each observation by a phenotype of interest that was nested within a given study: ANOVA(lmer(utility∼modality+(1|study/phenotype))) and lmer(utility∼dimensionality+(1|study/phenotype)). This change to the random intercept was necessary to account for the fact that some studies evaluated more than one phenotype. Lastly, a linear mixed-effects models was also used to evaluate the relationship between accuracy and utility: lmer(accuracy∼utility+(1|study/phenotype)).

## Results

### Characteristics of included studies

Significant heterogeneity in sample characteristics and methodological choices was observed across all twenty studies that met our inclusion criteria (Table 1). Specifically, a large number of studies used open-source neuroimaging datasets, with the most popular being from the Cambridge Center for Aging and Neuroscience dataset^51^ (n = 4) and the UK Biobank^52^ (n = 3). Most of the studies (n = 8) were trained and tested on cross-sectional samples with age ranges spanning from late adolescence to late adulthood (18–85 years). Datasets varied considerably in size, as the smallest comprised of 50 participants, while the largest contained 17,461. Additionally, a variety of phenotypes were used to evaluate the utility of these models, with the most common phenotypes relating to cognitive function and physical fitness. Lastly, there was considerable heterogeneity with respect to validation methods and predictive algorithms. Each study applied at least one of the following statistical models on neuroimaging data with the goal of continuously predicting age: seven used either regularized (e.g., least absolute shrinkage and selection operator),^53^ Ridge,^54^ ElasticNet,^55^ or non-regularized (e.g., ordinary least squares) linear regression;^56^ eight used support vector regression with a non-linear/radial basis kernel or tree-based algorithms (e.g., random forest);^57^ gradient tree boosting;^58^ five used a deep-learning framework (i.e., neural networks);^59^ and three used an ensemble modeling or model stacking approach^60^ (Table 1).

Each of these twenty studies used different imaging modalities and neural properties (Table S2). Briefly, all studies extracted anatomical properties derived from T1-weighted MRI scans, such as brain volume, cortical thickness, and surface area. Diffusion-weighted imaging (DWI) was the second most common modality from which indices of brain microstructure were computed using diffusion tensor imaging^61^ (DTI) or neurite orientation and dispersion density imaging^62^ (NODDI). The third most common imaging modality was resting-state fMRI, except for one study that also evaluated task-based MRI wherein participants engaged in an emotional face matching task.^63^ Functional connectivity was the most commonly extracted feature from fMRI scans, as only two studies evaluated regional homogeneity^64^ or the amplitude of low-frequency fluctuations.^65^ Some additional modalities that were less common included T2-weighted MRI, arterial spin-labeling MRI, and MEG. Nine out of these twenty studies compared brain features from three or more imaging modalities.

### Aim 1: Multimodal imaging improves the accuracy of brain age models

**Figure 2:**
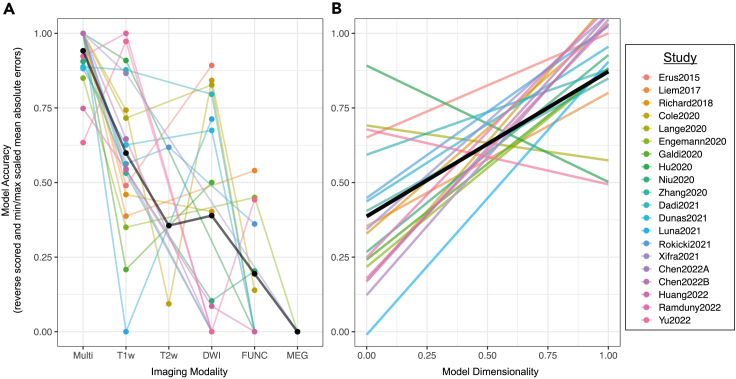
The most accurate brain age models were trained with multiple modalities and the greatest number of features possible (A) The multimodal models were the most accurate in all but two studies. The second and third most accurate brain age models were derived exclusively from T1-weighted and DWI-weighted features, respectively. (B) Model accuracy varied as a function of the number of features used to train each model. The three studies that were outliers in this scatterplot could be attributed to their use of highly dimensional functional connectivity features, which were not as accurate as brain morphometry or white-matter microstructure. Scaled mean absolute errors (MAEs) with a score of 1 were the most accurate within a given study, while those with a 0 were the least accurate. Each colored line represents the average of all models from a specific study, while the back line represents all models across all studies.

Collectively, 117 models were developed across all twenty brain age studies, with each model being derived from a distinct set of neuroimaging data. Across this body of literature, the multimodal models were the most accurate (Figure 2A; Table S2) compared with all other groups of models that were derived from a single imaging source (β > 0.24, p < 0.002). This converging finding was supported by analyzing differences within each study, as the multimodal models were most accurate in all but two cases; even so, in those two atypical cases, the multimodal models were only slightly less accurate than the anatomical models derived solely from T1-weighted scans.^39^^,^^40^ Further, training brain age models with the greatest number of features yielded the lowest prediction errors (β = 0.52, p < 0.001; Figure 2B), and this association was not moderated by sample size or imaging modality (Figure S2). In cases when less accurate or “noisy” features were included, the accuracy of brain age models did not decline.^26^ However, adding more features from a single modality did not benefit model accuracy as much as adding complimentary information from another modality.^24^ Taken together, these results indicate that both incorporating multiple modalities and, to a lesser extent, increasing the number of features were essential for improving a model’s capacity to accurately predict age.

Although the multimodal brain age models were most accurate, certain modalities had a disproportionate impact on the predictive power of brain age models. Specifically, models trained on anatomical features (i.e., cortical thickness and volume) or white matter microstructure (i.e., mean diffusivity and apparent diffusion coefficient) were consistently more accurate than those derived from markers of brain activation (i.e., amplitude of low frequency fluctuations) or functional organization (i.e., functional connectivity; Figure 2A). Further, most of the studies included in this review (n = 12) performed feature importance analyses to illustrate which individual regions had a significant contribution to brain age predictions. Across these analyses, the features that consistently had the largest importance were morphometric measurements of the precuneus, orbitofrontal cortex, and deep brain structures. Specifically, the predictive power of brain age models could be most attributed to the thalamus, striatum, cingulate, insula, ventricles, hippocampus, caudate, and amygdala.^12^^,^^35^ Additionally, the white matter tracts that contributed the most to the predictive power of brain age models included the corpus callosum, choroid plexus, fornix, and uncinate fasciculus.^2^^,^^26^^,^^34^ Therefore, the most accurate unimodal brain age models appeared to be those that were based on anatomical, as opposed to functional, MRI properties.

Another dimension of model accuracy is age-related prediction bias, which is a well-known limitation of the brain age framework.^19^ This bias refers to the fact that brain ages of younger individuals are overestimated while the predictions of older individuals are underestimated.^44^ Nearly all unimodal models are universally affected by this bias,^12^ but it is unclear whether multimodal models are also susceptible. Through our review of the multimodal literature, we identified only three models whose brain age predictions were not systematically biased, as their brain age gaps did not vary as a function of age.^2^^,^^35^^,^^66^ A commonality between these three models was that they all applied traditional machine-learning models to spatial maps from structural MRI sequences, primarily T1-weighted and diffusion-weighted scans. However, other studies used similar approaches and obtained models whose predictions were still susceptible to biases.^23^^,^^32^ Therefore, it is still not robustly clear whether using multimodal imaging or certain feature types can mitigate issues of prediction bias.

### Aim 2: Model utility depends on both imaging modality and the phenotype of interest

**Figure 3:**
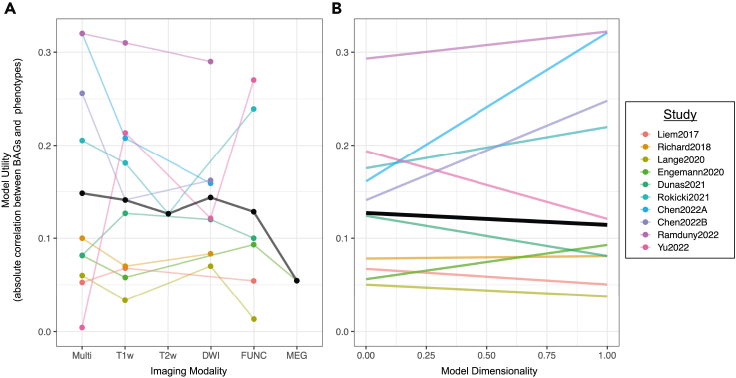
The most useful brain age models varied from study to study (A) Model utility varied considerably across studies, such that no single set of features emerged as being substantially more useful. (B) The utility of brain age models did not vary as a function of the number of features used to train them. Each colored line represents the average across all models from a specific study, while the back line represents all models across all studies.

Among the ten studies that compared the utility of multiple brain age models, a total of 55 models were developed and evaluated using 13 distinct phenotypes (Figure S3). Similar to our assessment of model accuracy, the multimodal brain age models were the most useful across all studies, but such differences were only statistically significant when compared with the models trained with features derived solely from T2-weighted or MEG scans (β > 0.22, p < 0.002; Figure 3A). Further, the utility of all brain age models did not differ by the number of neuroimaging features upon which they were trained (β = 0.02, p = 0.75; Figure 3B). It is possible that significant differences in model utility did not emerge (relative to model accuracy) because of the heterogeneity in phenotypes of interest and discrepancies in the methods used to quantify them.

To understand whether the utility of brain age models depended on the phenotype in question, we narrowed our focus on two studies that contained the largest number of phenotypes. Rokicki et al.^34^ demonstrated that nearly all brain age models were able to detect differences in Alzheimer’s disease and schizophrenia. However, brain age models that were derived from either multimodal features or strictly morphometric features from T1-weighted MRI scans yielded the largest effect sizes. In contrast, models that were based on cerebral blood flow from arterial spin-labeling MRI scans were the only ones to be significantly associated with all five of their phenotypes, including bipolar disorder, cognitive complaints, and mild cognitive impairments. These findings partially aligned with Engemann et al.,^27^ who reported that multimodal models yielded the strongest effect sizes, whereas models derived exclusively from brain activation (via MEG) and functional connectivity (via fMRI) were related to certain phenotypes that other models could not detect. Taken together, models derived from multimodal or morphometric brain features appear to yield the strongest correlations among a limited number of phenotypes, whereas those derived from functional imaging modalities were sensitive to the largest number of phenotypes.

**Figure 4:**
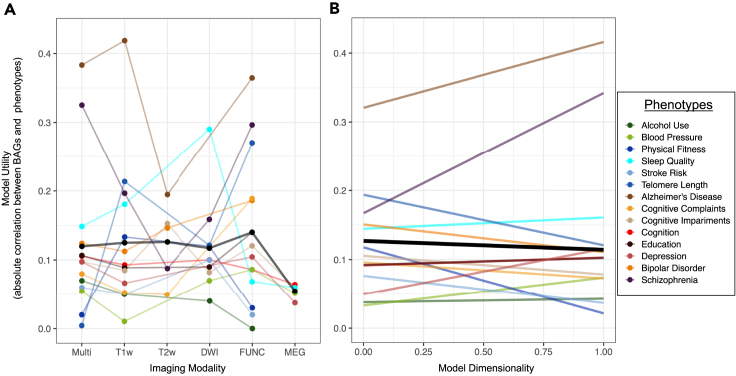
The utility of brain age models differed between phenotype of interest (A) The fMRI brain age models exhibited a slight advantage in utility. However, multimodal models yielded the strongest effect sizes when detecting differences in chronic brain disorders, including Alzheimer’s disease and schizophrenia. (B) Similarly, brain age models that were derived with a greater number of features were generally better at detecting differences in chronic brain disorders. Each colored line represents the average across all models from a specific study, while the back line represents all models across all studies.

**Figure 5:**
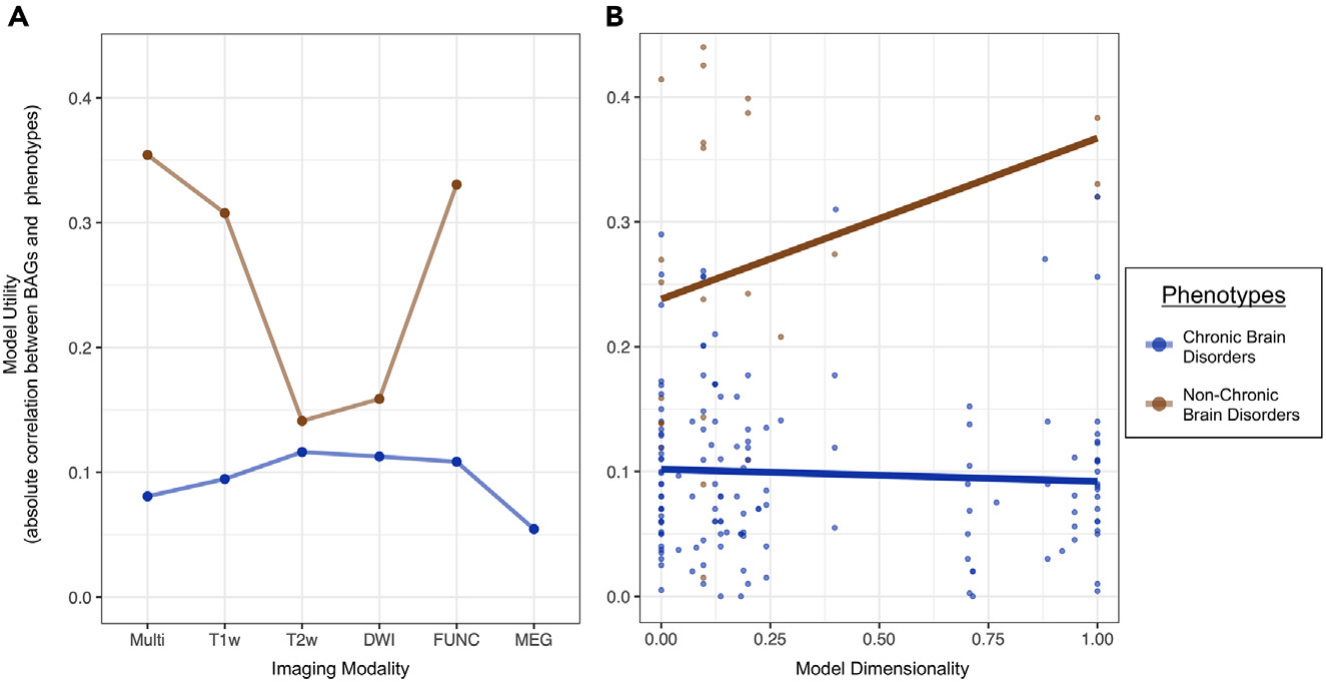
Brain age models were best at detecting differences in chronic brain disorders (A) Brain age models were best at detecting differences in Alzheimer’s disease and schizophrenia. These improvements in model utility were most prominent when using multimodal, anatomical, or functional MRI features. (B) As the number of features increased, brain age models were better at detecting differences in these chronic brain conditions, but this was not the case when grouping together all twelve of the remaining phenotypes.

To further investigate whether the utility of brain age models depends on the phenotype in question, we assessed these relationships across all ten studies using a quantitative synthesis approach.^18^ As expected, Alzheimer’s disease and schizophrenia exhibited the strongest effect sizes (Figure 4A), especially for the multimodal, T1-weighted, or functional brain age models. In contrast, all other phenotypes exhibited weaker associations that were best detected by the functional brain age models. These dynamics were also reflected in the relationship between model utility and the number of features used to train each model (Figure 4B). Specifically, brain age models that used the largest number of features were more able to detect differences in Alzheimer’s disease and schizophrenia, but these relationships were stable or decreasing for almost all other phenotypes. Therefore, it appears that model utility depends on both the phenotype of interest and neuroimaging features used (Figure 5), but not all studies followed this emerging pattern. In particular, Lange et al.^26^ reported that models based on functional connectivity were the least useful at detecting differences in alcohol use, blood pressure, and stroke risk. It is worth noting that this study was conducted on one of the oldest cohorts, spanning from 60 to 85 years of age, which may partly explain why their results were relatively idiosyncratic.

### Aim 3: The accuracy and utility of brain age models are not related

**Figure 6:**
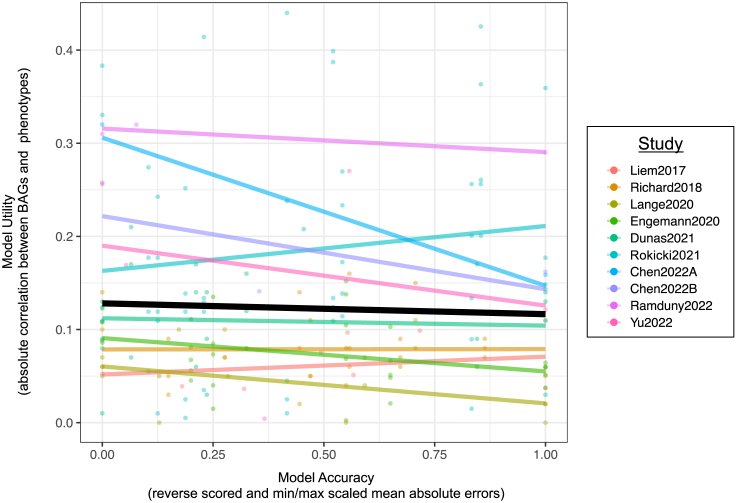
The utility of brain age models did not vary as a function of their accuracy Each point represents the accuracy and utility of a given model, which is color coded by the study it was gathered from. The black line denotes the relationship between accuracy and utility across all models from all ten studies. Scaled MAEs with a score of 1 were the most accurate within a given study, while those with a 0 were the least accurate.

Our review suggests that multimodal brain age models are almost always the most accurate but are not necessarily the most sensitive to differences across a variety of phenotypes. Such finding aligns with a developing narrative in the brain age literature that the accuracy and utility of brain age models are two unrelated performance metrics.^16^^,^^17^ Mixed-effects models were used to further evaluate this notion across all brain age models from the ten included studies in this review. As expected, the utility of these models did not vary as a function of their accuracy (β = −0.02, p = 0.73; Figure 6). This result was further supported when evaluating each study individually, as the data from one study suggested that model utility was better when accuracy was worse (β = −0.54, p = 0.04),^26^ and all other studies did not display any substantial trends or associations (β < −0.33, p > 0.07).

## Discussion

This systematic review demonstrated that feature selection has a large downstream impact on both the accuracy and utility of brain age models. Yet, we could not identify a single set of neuroimaging features that led to uniform improvements across all performance metrics and conditions, indicating that unimodal and multimodal models each have distinct use cases. Indeed, these findings have been echoed in prior studies. Liem et al.^24^ was one of the first studies to conclude that multimodal models were best at predicting age, while specific imaging modalities were superior at detecting differences in pathology. Despite mounting evidence, there is a persisting assumption across several studies that the most accurate brain age models will have the most potential for detecting differences in a given phenotype of interest. As a point of illustration, seven of the twenty studies in this review only evaluated the utility of their most accurate model, which in all cases was trained using multimodal features. This approach has also led to researchers to exclusively use T1-weighted and diffusion-weighted MRI scans when developing brain age models^36^ since such modalities have been shown to have the largest contribution to a model’s predictive power.^2^^,^^67^ However, our review suggests that model accuracy does not necessarily provide meaningful insight about clinical utility (e.g., detection of age-related pathology). Taken with prior studies,^16^^,^^17^ it appears that the most accurate models tend to not be the most useful. Yet, it is not robustly clear how accurate brain age predictions should be to increase a model’s capacity for detecting individual differences.^68^ These results highlight that a more exhaustive approach may be necessary when making comparisons between brain age models. For instance, evaluations of brain age models should go beyond comparing mean absolute errors and also evaluate their models’ utility across a variety of phenotypes. Given that our results regarding model utility were relatively more variable across studies and based on a limited number of observations (Figure S4), we believe that further evaluating the clinical applications of unimodal vs. multimodal models remains a top priority.

Additionally, we observed that the utility of brain age models largely depended on the phenotype of interest. In particular, brain age models were best able to explain differences in chronic brain disorders, including Alzheimer’s disease and schizophrenia.^34^ We propose three potential reasons as to why these specific phenotypes yielded the strongest correlations with brain age gaps. First, Alzheimer’s disease and schizophrenia are progressive in nature, as the onset and severity of symptoms are robustly associated with age.^69^^,^^70^ Secondly, both forms of pathology disrupt multiple facets of cognition and mental health, which may be easier to quantify or diagnose.^71^ Lastly, Alzheimer’s disease and schizophrenia have been shown to have severe and widespread impacts on the brain,^72^^,^^73^ potentially making brain age prediction more sensitive to disruptions associated with these two illnesses. Further, the widespread brain atrophy associated with each of these illnesses is reflected by a considerable shrinkage in brain volume,^74^ which is a feature type that is heavily weighted by multimodal brain age models. Altogether, these factors may each contribute to patients with Alzheimer’s disease and schizophrenia having larger brain age gaps that widen overtime.^42^^,^^75^ Nevertheless, it could be beneficial for additional studies to disentangle which of these three possibilities most substantially improved the utility of brain age models.

Although Alzheimer’s disease and schizophrenia were best captured by multimodal brain age models, it appeared that these models were not as useful in detecting differences in other phenotypes. Our interpretation of these results is that multimodal brain age models most heavily weigh the features that can accurately predict age, regardless of their clinical relevance. As a consequence, some features with little clinical significance still had an outsized contribution to brain age predictions, including the corpus callosum, choroid plexus, lateral ventricles, and cerebellum.^76^ In contrast, numerous subcortical structures of the limbic system were also heavily weighted by brain age models, including the thalamus, hippocampus, and amygdala.^12^^,^^35^ Each of these structures plays a crucial role in facilitating cognitive functioning^77^ and emotional regulation.^78^ Further, abnormalities in the morphometric measurements of hippocampus and amygdala are robust biomarkers of both Alzheimer’s disease^72^ and schizophrenia.^43^ These findings may help to decode why multimodal models were sensitive to these chronic brain disorders, but ways to systematically improve their utility to detect other phenotypes remain unclear.

Aside from chronic brain disorders, all other phenotypes were robustly associated with brain age models that were derived solely from fMRI features. Nearly all the included studies reported that features of brain activation or functional organization encoded unique information that was useful in detecting individual differences.^27^^,^^34^ However, when functional features were combined with metrics of brain morphometry or white-matter microstructures, we observed a reduction in model utility. This consequence might have occurred because multimodal brain age models more heavily prioritize structural features due to their robust associations with age and reliability across the lifespan.^35^ Although functional brain features have a limited capacity to accurately predict age, they appear to be more sensitive to transitory life events or state-like phenotypes.^26^ It is possible that fMRI features were more sensitive to earlier indicators of pathology because they are more malleable and likely to precede changes in brain structure.^79^ Taken further, these precedents suggest that fMRI features could be more useful in predicting pathology directly. Future longitudinal studies are needed to determine how structure and function dynamics across time may lead to differences in the utility of these machine-learning frameworks. Altogether, fMRI features appear to have the most potential for developing brain age models with widespread utility.

This systematic review contains several limitations. Accounting for all unwanted sources of variation when making inferences between brain age studies can be challenging because of differences in sample characteristics and methodological decisions.^41^ We addressed these challenges by min/max scaling the mean absolute errors and the number of features used for each model from a given study, thereby placing emphasis on within- and across-study differences. Therefore, our conclusions that are based on these standardized metrics may be confounded by differences between studies, such as sample demographics, clinical phenotypes, social factors, data quality, model complexity, and regularization techniques. For example, differences in regularization techniques can largely influence the accuracy and utility of brain age models as well as the stability of the model weights.^15^ Further, our quantitative synthesis was limited in statistical power, especially when evaluating the utility for a given set of neuroimaging features by specific phenotypes of interest. At times, we only had access to the results of a single study, such as those from Rokicki et al.,^34^ which was the only multimodal study to evaluate Alzheimer’s disease, bipolar disorders, and cognitive complaints. Given these limits in statistical power, it was not feasible to employ formal meta-analysis methods like those used for quantifying the heterogeneity between studies.^80^ Nevertheless, it was encouraging that our findings overlapped with unimodal brain age studies that were not included in this review, but also demonstrated that brain age gaps were most sensitive to differences in chronic brain disorders, including Alzheimer’s disease and schizophrenia.^6^^,^^42^

Since the first multimodal brain age study was published in 2011, the enthusiasm for using multimodal imaging has only increased. Theoretically, providing brain age models with complimentary information about brain development should result in more nuanced predictions that can better reflect individual differences. In practice, the primary advantage of multimodal models was an increased capacity to predict age, which did not translate to widespread improvements in their utility. These conclusions build upon prior demonstrations that the most accurate models tend to not be the most useful.^16^^,^^17^ Additionally, it could be argued that providing brain age models with a larger feature space makes them more susceptible to issues of overfitting.^81^ Taken together, we believe that more creative approaches are needed to further maximize the benefits that brain age models could leverage from multimodal imaging. One possible avenue could be to apply a fitness function to a set of brain age gaps with the goal of maximizing the separability between individuals based on a selected distance criterion. This approach has been shown to improve sensitivity to Alzheimer’s disease^82^ and could have greater implications if applied to multimodal models. However, researchers must consider whether the added benefits of multimodal brain age models outweigh the costs of limited transferability.^83^^,^^84^^,^^85^ Specifically, more data will need to be collected for a multimodal model to be applied, which is not always feasible, especially in clinical settings. Further, more work is needed to evaluate higher-order features of brain networks. For example, the edge strength was the most commonly used fMRI feature across this body of literature. It is unknown whether indices of graph theory^86^ or network cohesion^87^ could lead to even greater improvements in model utility. To conclude, multimodal imaging has provided us with valuable insight for improving the accuracy of brain age models, but there is still much untapped potential in regard to achieving widespread clinical utility.

## Supplemental information

Document S1. Figures S1–S4 and Tables S1–S3

Document S2. Article plus supplemental information
