# Supplementary material for: A systematic review of multimodal brain age studies: Uncovering a divergence between model accuracy and utility
Source: Patterns (N Y). 2023 Apr 14;4(4):100712. doi: 10.1016/j.patter.2023.100712 (PMC10140612; doi:10.1016/j.patter.2023.100712)
Supplement: Document S1. Figures S1–S4 and Tables S1–S3 [file mmc1.pdf]

Patterns, Volume *4*

Supplemental information

A systematic review of multimodal brain age studies: Uncovering a divergence between model accuracy and utility

Robert J. Jirsaraie, Aaron J. Gorelik, Martins M. Gatavins, Denis A. Engemann, Ryan Bogdan, Deanna M. Barch, and Aristeidis Sotiras

SUPPLEMENTAL MATERIAL

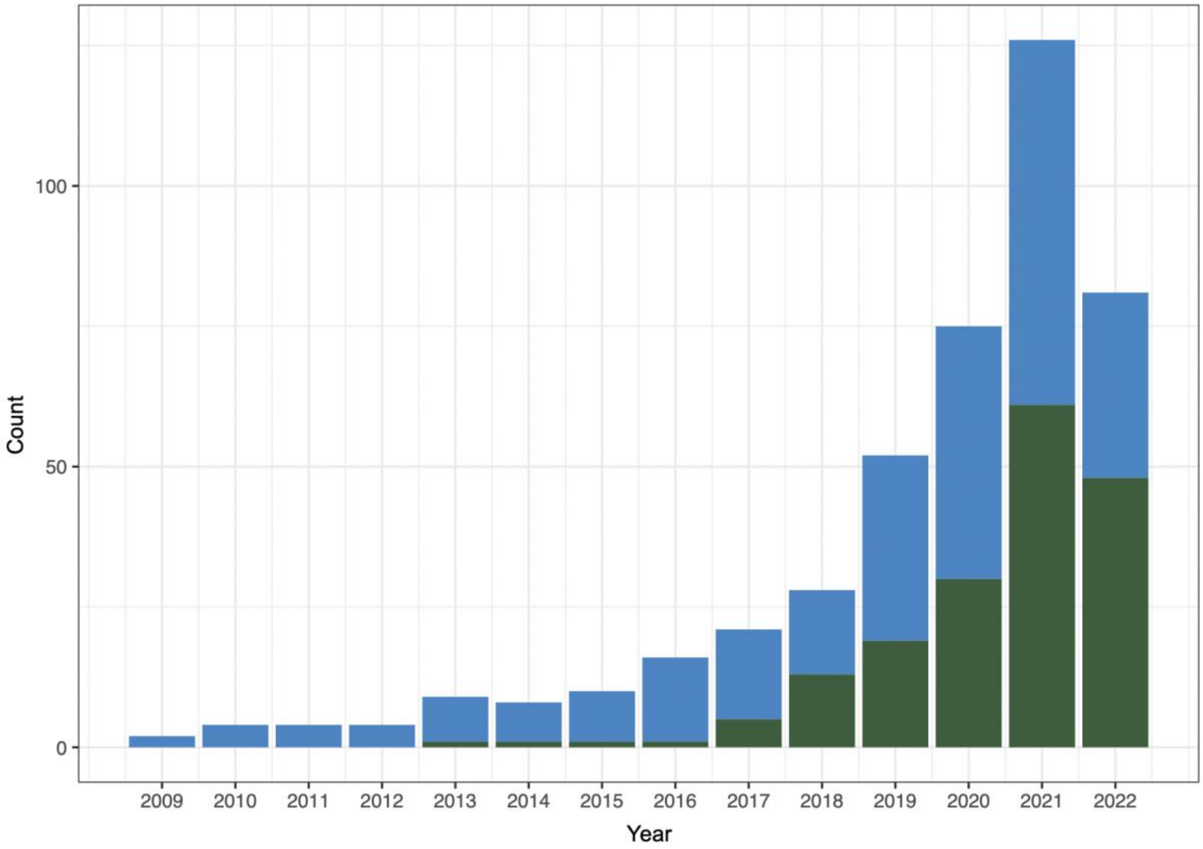

Figure S1. The proportion of brain age articles that contain the word "multimodal" increases over time. This histogram shows the total number of articles published in a given year that used the term "brain age" in their title, abstract, or keywords. The green shaded bars are the portion of brain age studies that either mentioned or referenced the term "multimodal" anywhere throughout the article. The blue shaded bars represent the brain age articles that did not use the term multimodal.

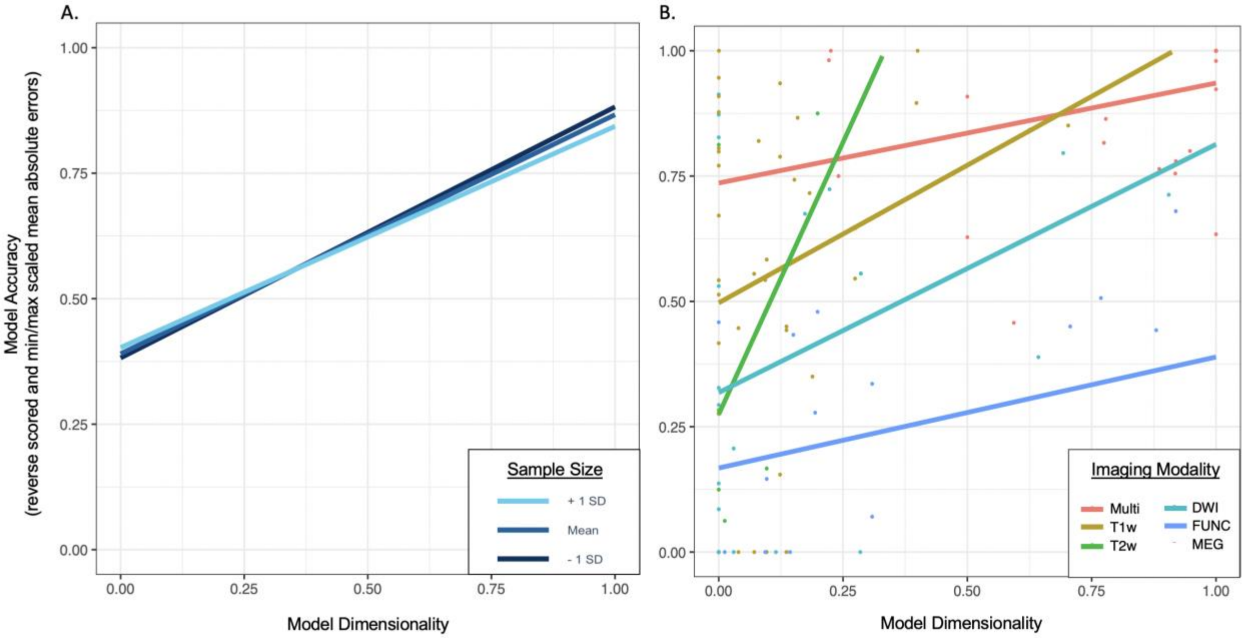

Figure S2. The relationship between model accuracy and number of features was not moderated by sample size or imaging modality. A. Brain age models with a greater number of neuroimaging features tend to have better accuracy regardless of the sample size used to train and evaluate them ($\beta = -0.05$, $p = 0.71$). **B.** When evaluating models within a given imaging modality, model accuracy was consistently correlated with the number of features used. Scaled MAEs with a score of 1 were the most accurate within a given study while those with a 0 were the least accurate.

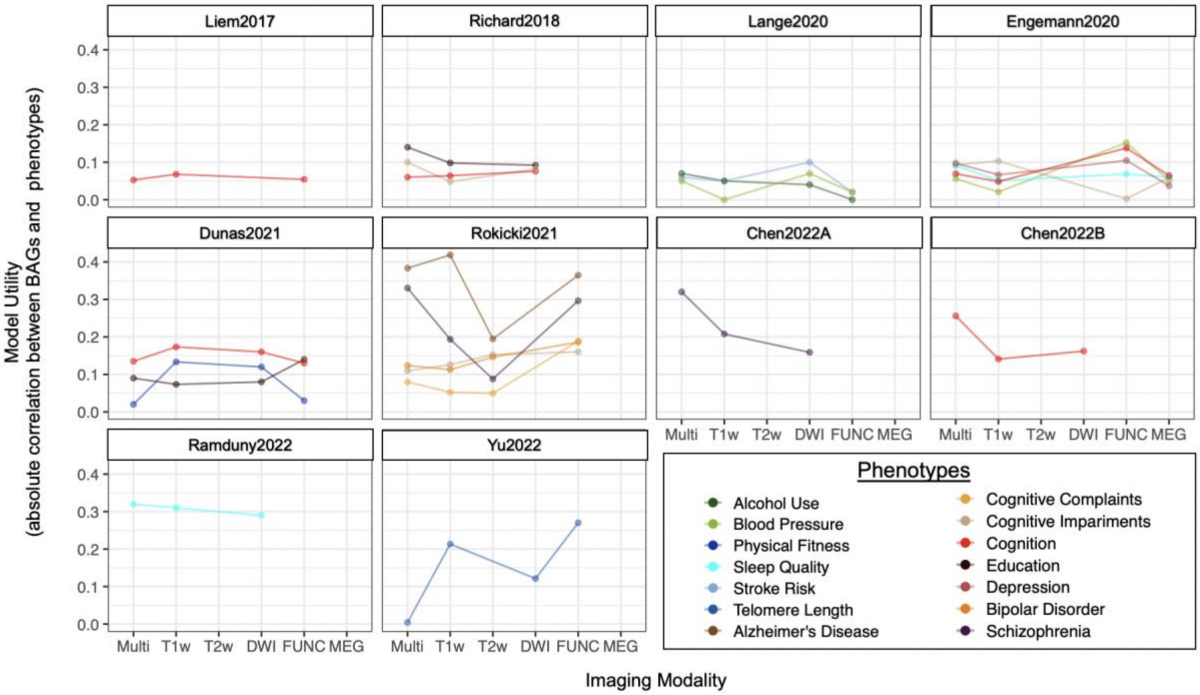

Figure S3. The utility of brain age models varied considerably by the phenotype of interest. When evaluating studies that analyzed multiple phenotypes it became apparent that the utility of brain age models was largely dependent on the phenotype in question. Each colored line represents the utility of brain age models for a specific phenotype.

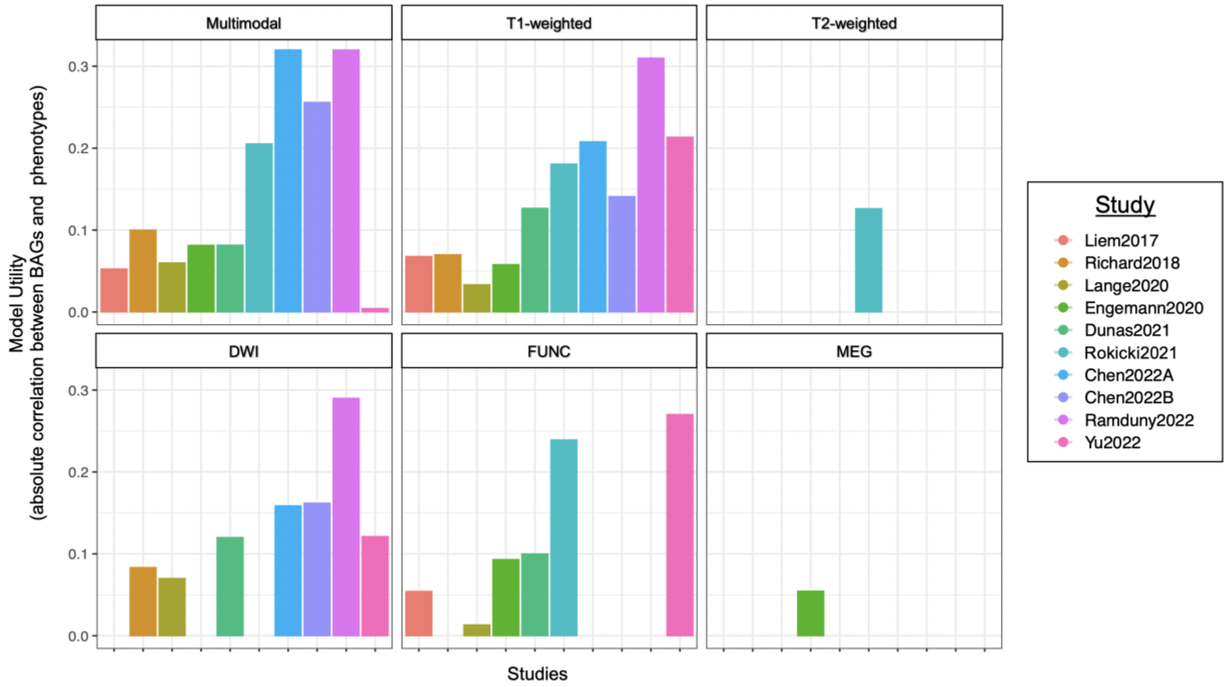

Figure S4. The utility of brain age models varied considerably between imaging modalities and across studies. Each bar denotes that the utility of brain age models that were derived from a given imaging source for a specific study. There was considerably heterogeneity between in model utility across the ten studies included in this review.

Table S1. Neuroimaging Modalities and Feature Types.

Study	Structural MRI	Functional MRI	Diffusion MRI	Miscellaneous
Erus et al. (2015)[maps]	CT, WM, VN	-	FA, ADC	-
Liem et al. (2017)	CT, SA, subcortical volume	rsFC (197, 444)	-	-
Richard et al. (2018)	CT, SA, CVol	-	FA, MD, RD, AD	-
Cole (2020)	CT, SA, CVol	rsFC, taskFC	FA, MD, MO, L1:3, ICVF, OD, ISOVF	T2-FLAIR, T2*
de Lange et al. (2020)	CT, SA, CVol	rsFC (ICA, PCorr)	FA, MD, AD, RD, MO	global WMH volume
Engemann et al. (2020)	CT, SA, subcortVol	rsFC	-	MEG
Galdi et al. (2020)	Regional volumes	-	FA, MD, AD, RD, MK, ICVF, ISOVF, OD, OD1, OD2	T1w/T2w ratio, constructed MSN
Hu et al. (2020)	LGI, MC, AC, CT, SA, CVol	rsFC	-	-

Niu et al. (2020)	CVol	ALFF, ReHo	FA, MD	-
Zhang et al. (2020)	CT, SA, CVol	-	connectome	-
Dadi et al. (2021)	CVol, subcortical volume	rsFC (ICA)	FA, MD, MO, ICVF, ISOVF, OD	-
Dunas et al. (2021)[maps]	CVol maps, WMVol, CSF maps	rsFC	FA intensity map	-
Luna et al. (2021)	CT, CVol, SA, MC	-	connectome	-
Rokicki et al. (2021)	CT, SA, CVol	-	-	T1w/T2w signal ratio, CBF
Xifra-Porxas et al. (2021)[maps]	GM, WM, CSF	-	-	MEG maps
Chen et al. (2022a)	CVol, CT	-	FA, MD	-
Chen et al. (2022b)	CVol, CT	-	FA, MD	-
Huang et al. (2022)	CVol, WM Vol	ALFF, ReHo, rsFC DC	FA, MD, RD, AD	-
Ramduny et al. (2022)	CVol, subcortical volume	-	FA, MD	-

Yu et al. (2022)	CT, subcortical volume	rsFC	connectome	-

Note. maps – maps used to denote that spatial maps were the input features used for a given predicative algorithm.

Structural (T1w) MRI measures: CVol (cortical gray matter volume), WMVol (white matter volume), VNVol (ventricle volume), CT (cortical thickness), SA (cortical surface area), LGI (local gyrification index), MC (mean curvature), AC (average convexity), CSF (cerebrospinal fluid intensity)

Functional MRI measures: rsFC (resting-state functional connectivity): ICA (defining nodes using independent component analysis), PCorr (using partial correlations between time series at different parcellated regions); ALFF (amplitude of low-frequency fluctuations), ReHo (regional homogeneity), DC (degree centrality in a functional connectivity network), taskFC (task-based MRI functional connectivity)

Diffusion weighted imaging measures: FA (fractional anisotropy), MD (mean diffusivity), RD (radial diffusivity), AD (axial diffusivity), ADC (apparent coefficient of diffusion), ICVF (intracellular volume fraction), ISOVF (isotropic volume fraction), MO (mode of anisotropy), MK (mean kurtosis), OD (total orientation dispersion), OD1 & OD2 (orientation dispersion along primary (1) and secondary (2) axes), L1-3 (diffusion tensor along primary, secondary, and tertiary axes of an ellipse), Connectome (tractography-derived structural connectivity matrix).

Other measures: CBF (cerebral blood flow), MSN (morphometric similarity networks), WMH (white matter hyperintensities)

Table S2. Accuracy of brain age models from all twenty studies included in this review.

Study	Multimodal (MAE | r | COD)	T1-weighted MRI (MAE | r | COD)	T2-weighted MRI (MAE | r | COD)	Diffusion MRI (MAE | r | COD)	Functional MRI (MAE | r | COD)	MEG (MAE | r | COD)
Erus et al. (2015)$_{maps}$	1.00 | 0.88 | 0.79	0.49 | 0.69 | 0.47	-	0.98 | 0.84 | 0.71	-	-
Liem et al. (2017)	1.00 | NA | NA	0.39 | NA | NA	-	0.54 | NA | NA	-	-
Richard et al. (2018)	1.00 | 0.85 | 0.73	0.46 | 0.73 | 0.53	-	0.40 | 0.71 | 0.50	-	-
Cole (2020)	1.00 | 0.78 | 0.62	0.74 | 0.69 | 0.47	0.09 | 0.32 | 0.10	0.84 | 0.73 | 0.53	0.14 | 0.33 | 0.11	-
de Lange et al. (2020)	1.00 | 0.54 | 0.30	0.72 | 0.47 | 0.22	-	0.83 | 0.49 | 0.24	0.00 | 0.00 | 0.00	-
Engemann et al. (2020)	0.85 | NA | NA	0.35 | NA | NA	-	-	0.45 | NA | NA	0.00 | NA | NA
Galdi et al. (2020)	1.00 | 0.78 | 0.61	0.21 | 0.47 | 0.22	-	0.50 | 0.58 | 0.34	-	-
Hu et al. (2020)	1.00 | NA | NA	0.91 | NA | NA	-	-	0.00 | NA | NA	-
Niu et al. (2020)	0.91 | 0.87 | 0.75	0.54 | 0.82 | 0.67	-	0.10 | 0.76 | 0.58	0.20 | 0.77 | 0.60	-
Zhang et al. (2020)	1.00 | NA | NA	0.53 | NA | NA	-	0.00 | NA | NA	-	-
Dadi et al. (2021)	0.89 | NA | NA	0.88 | NA | NA	-	0.80 | NA | NA	0.00 | NA | NA	-
Dunas et al. (2021)$_{maps}$	0.88 | NA | NA	0.62 | NA | NA	-	0.67 | NA | NA	0.00 | NA | NA	-
Luna et al. (2021)	1.00 | NA | NA	0.00 | NA | NA	-	0.71 | NA | NA	-	-

Rokicki et al. (2021)	1.00 | 0.87 | 0.77	0.56 | 0.76 | 0.58	0.62 | 0.78 | 0.61	-	0.36 | 0.69 | 0.47	-
Xifra-Porxas et al. (2021)$_{maps}$	1.00 | NA | NA	0.87 | NA | NA	-	-	-	0.00 | NA | NA
Chen et al. (2022a)	1.00 | 0.97 | 0.94	0.54 | 0.96 | 0.94	-	0.00 | 0.94 | 0.88	-	-
Chen et al. (2022b)	1.00 | 0.89 | 0.80	0.65 | 0.87 | 0.76		0.00 | 0.76 | 0.58	-	-
Huang et al. (2022)	0.75 | 0.84 | 0.70	0.54 | 0.79 | 0.62	-	0.08 | 0.72 | 0.52	0.00 | 0.70 | 0.49	-
Ramduny et al. (2022)	0.92 | 0.74 | 0.54	1.00 | 0.76 | 0.58	-	0.00 | 0.64 | 0.41	-	-
Yu et al. (2022)	0.63 | 0.54 | 0.29	0.97 | 0.55 | 0.30	-	0.00 | 0.52 | 0.27	0.44 | 0.46 | 0.21	-

Note. Evaluation metrics regarding the accuracy of brain age models are reported. If a given study evaluated multiple models derived from a single imaging modality then the metrics across all model variants were averaged. To quantify mean absolute error, we first min/max normalized the prediction errors from within a given study, and subsequently, inverted them. A score of 1 suggests that models from these feature types were the most accurate within a given study, whereas a score of 0 denotes that the least accurate models within a given study. Therefore, this definition of model accuracy most reflected relative performance within a given study as opposed to differences between studies. The second and third accuracy metrics reported in each table cell is the correlation and coefficient of determination, respectively. Such accuracy metrics were recorded if the original study reported the Pearson correlation between chronological and brain ages.

Table S3. Utility of brain age models from a subset of ten studies included in this review.

Study	Multimodal	T1-weighted MRI	T2-weighted MRI	Diffusion MRI	Functional MRI	MEG
Liem et al. (2017)	0.05	0.06	-	0.05	-	-
Richard et al. (2018)	0.10	0.07	-	0.08	-	-
de Lange et al. (2020)	0.06	0.03	-	0.07	0.01	-
Engemann et al. (2020)	0.08	0.06	-	-	0.09	0.05
Dunas et al. (2021)$_{maps}$	0.08	0.13	-	0.12	0.10	-
Rokicki et al. (2021)	0.21	0.18	0.13	-	0.23	-
Chen et al. (2022a)	0.32	0.21	-	0.16	-	-
Chen et al. (2022b)	0.26	0.14		0.16	-	-
Ramduny et al. (2022)	0.32	0.31	-	0.29	-	-
Yu et al. (2022)	0.01	0.21	-	0.12	0.27	-

Note. Evaluation metrics regarding the utility of brain age models are reported. If a given study evaluated multiple models derived from a single imaging modality then the metrics across all model variants were averaged. Model utility was quantified as the absolute value of the correlation between brain age gaps and phenotypes of interest. Taking the absolute value was preferred to more broadly reflect the strengthen of the effect size across multiple phenotypes.
