# Supplementary material for: A systematic review of multimodal brain age studies: Uncovering a divergence between model accuracy and utility
Source: Patterns (N Y). 2023 Apr 14;4(4):100712. doi: 10.1016/j.patter.2023.100712 (PMC10140612; doi:10.1016/j.patter.2023.100712)
Supplement: Document S2. Article plus supplemental information [file mmc2.pdf]

Patterns

CellPress
OPEN ACCESS

Review

# A systematic review of multimodal brain age studies: Uncovering a divergence between model accuracy and utility

Robert J. Jirsaraie,[1] Aaron J. Gorelik,[2] Martins M. Gatavins,[1,3] Denis A. Engemann,[4,5] Ryan Bogdan,[2] Deanna M. Barch,[2] and Aristeidis Sotiras[6,*]

[1]Division of Computational and Data Sciences, Washington University in St. Louis, St. Louis, MO, USA
[2]Department of Psychological & Brain Sciences, Washington University in St. Louis, St. Louis, MO, USA
[3]Undergraduate Neuroscience Program, School of Arts & Sciences, University of Pennsylvania, Philadelphia, PA, USA
[4]Roche Pharma Research and Early Development, Neuroscience and Rare Diseases, Roche Innovation Center Basel, F. Hoffmann-La Roche, Ltd., Basel, Switzerland
[5]Université Paris-Saclay, Inria, CEA, Palaiseau, France
[6]Department of Radiology and Institute for Informatics, Washington University School of Medicine in St. Louis, St. Louis, MO, USA
*Correspondence: aristeidis.sotiras@wustl.edu
https://doi.org/10.1016/j.patter.2023.100712

**THE BIGGER PICTURE** Establishing standardized biomarkers of brain aging would have immense utility for quantifying the risks of adverse life outcomes, which may lead to more precise and personalized interventions. An increasingly common approach for deriving such biomarkers is to train machine-learning models on a diverse set of neuroimaging features with the goal of accurately estimating a person's biological age. Further, the difference between biological and chronological ages has been shown to be a useful metric that is sensitive to many phenotypes. However, no definitive guidelines exist to help researchers decide which set of neural properties would improve model accuracy and utility. Accordingly, we conducted a systematic review of multimodal brain age studies to identify which neuroimaging features provided the largest added value. We found that the multimodal models had an increased capacity to predict age, which did not translate to widespread improvement in clinical utility.

1 2 3 **4** 5 **Production:** Data science output is validated, understood, and regularly used for multiple domains/problems

## SUMMARY

Brain aging is a complex, multifaceted process that can be challenging to model in ways that are accurate and clinically useful. One of the most common approaches has been to apply machine learning to neuroimaging data with the goal of predicting age in a data-driven manner. Building on initial brain age studies that were derived solely from T1-weighted scans (i.e., unimodal), recent studies have incorporated features across multiple imaging modalities (i.e., "multimodal"). In this systematic review, we show that unimodal and multimodal models have distinct advantages. Multimodal models are the most accurate and sensitive to differences in chronic brain disorders. In contrast, unimodal models from functional magnetic resonance imaging were most sensitive to differences across a broad array of phenotypes. Altogether, multimodal imaging has provided us valuable insight for improving the accuracy of brain age models, but there is still much untapped potential with regard to achieving widespread clinical utility.

## INTRODUCTION

Advances in computational resources and machine-learning architecture have empowered researchers to investigate brain aging in a data-driven manner.[1] It has become increasingly clear that applying machine learning to neuroimaging data can yield accurate estimates about the biological age of a person (i.e., brain age), which can serve as a biomarker for evaluating brain health.[2] Many studies have demonstrated that discrepancies between brain and chronological ages (i.e., brain age gaps) can be sensitive to differences across a variety of phenotypes, including cognitive functioning,[3] physical health,[4] mental disorders,[5] and biomedical conditions.[2] As such, brain age gaps can be useful for delineating many brain-behavior relationships, which we operationalize as "model utility." Given the broad implications of brain age models, it is essential to find ways of improving their

Check for updates


This is an open access article under the CC BY license (http://creativecommons.org/licenses/by/4.0/).

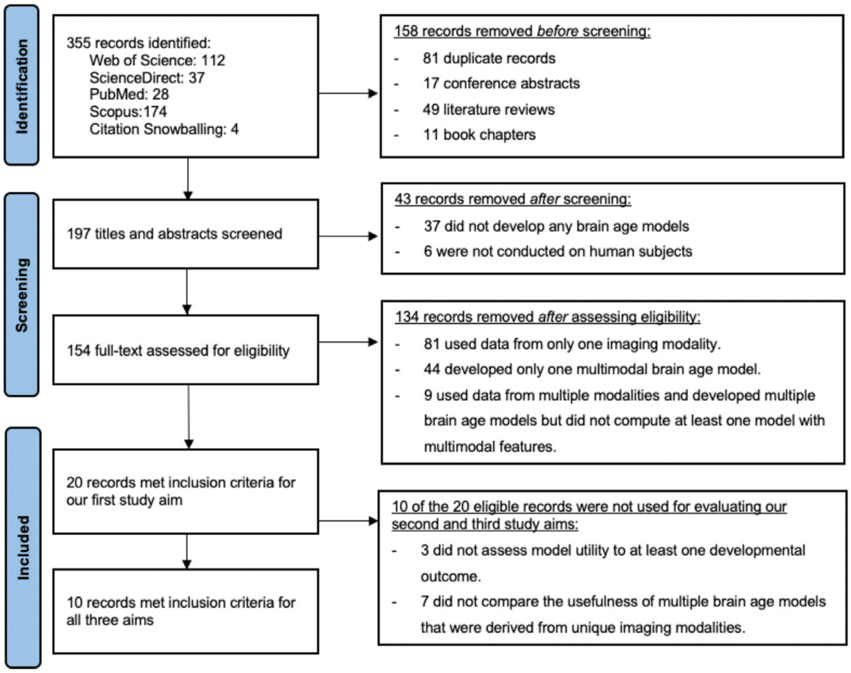


**Figure 1. Flow diagram depicting the identification, screening, and eligibility of the relevant studies for this systematic review**
Our literature searches uncovered 197 unique empirical records that were screened. Briefly, studies were excluded if they did not compare multiple brain age models using multiple imaging sources. A total of twenty studies met the inclusion criteria for our first study aim, and of these, ten met our inclusion criteria for all three aims.

clinical applicability such that they can be applied to assess the risks of atypical neurodevelopment and age-related neurodegeneration. One promising avenue for improving brain age models has been to combine features from multiple modalities and across distinct types of neural properties. By generating brain age models with complimentary information about how the brain alters over time (e.g., brain activation and connectivity), their predictions may capture more variation between individuals.[6] Motivated by the potential advantages that could be obtained from multimodal imaging, we conducted the first systematic review of multimodal brain age studies with a specific focus on evaluating their accuracy and utility.

The first multimodal brain age study was published on December 25, 2011, and incorporated data from three types of magnetic resonance imaging (MRI) sequences: T1-weighted, T2-weighted, and diffusion-weighting scans. Brown et al.[7] found that the neural features extracted from each of these modalities exhibited unique developmental trajectories throughout adolescences. More importantly, there was an alignment between the years when a given set of features exhibited the most age-related differences and the time when those features contributed the most to the predictive power of their multimodal brain age model. Further, their multimodal model explained more than 92% of the variance in age and yielded mean absolute errors that were as low as 1.03 years. The accuracy of this model underscored the potential advantages of using multimodal data. Since this initial report, there have been over 200 brain age-related articles that utilized or referenced the term "multimodal," a number that is increasing at an accelerated rate each year (Figure S1). It is expected that this trend will continue, as recent data-sharing requirements for NIH-funded projects[8] and open science initiatives[9] will provide easier access to large multimodal imaging datasets. Additionally, continual advancements in harmonization tools may enable researchers to aggregate even larger samples for training and validating brain age models.[10] These precedents demonstrate the relevance for a review about the potential benefits of using multimodal imaging, especially in the context of brain age modeling.

The brain age framework is an actively evolving field with a wide array of methods to select from for training and validating models. Specifically, there are numerous options to decide between the type of predictive algorithm, validation technique, imaging modality, preprocessing pipeline, spatial templates/atlases, and specific neuroimaging features to extract. Thus far, there are resources to help researchers in selecting predictive algorithms[11–13] and validation techniques[14,15] but no definitive guidelines regarding which neuroimaging modalities and neural properties to use. This is concerning because there is a vast number of neuroimaging-derived measurements available for both structural and functional data (e.g., cortical thickness, functional connectivity, mean diffusivity, etc.), thereby making it difficult to choose the best set of features to achieve a particular goal. Additionally, recent brain age studies have demonstrated that the most accurate models tend to not be the most useful at detecting differences in cognitive functioning.[16,17] Therefore, it is not only essential for researchers to be deliberate when selecting neuroimaging properties but also when selecting which performance metrics to use for evaluating their brain age model(s). To the best of our knowledge, there are no extensive guidelines to assist researchers in navigating these methodological decisions in a principled manner. Although this systematic review is unlikely to completely fill this gap in the literature, we believe that examining the potential benefits of multimodal brain age models would be a conducive starting point.

Through this systematic review, we investigated whether multimodal brain age models had improved accuracy and utility relative to models that were derived from a single imaging source (i.e., unimodal). We explored the following aims across all relevant studies using a quantitative synthesis approach.[18] We first evaluated which imaging modalities derived the most accurate brain age models (e.g., lowest prediction errors between chronological and brain ages) and whether model accuracy varied with the number of input features (aim 1). Qualitative assessments were conducted to further understand which specific feature types and regions of interest most contributed to the predictive power of brain age models. Additionally, we identified several brain age models whose predictions did not significantly vary with age, which is a well-known issue referred to as prediction

CellPress
OPEN ACCESS

**Table 1. Study design and sample characteristics**

| Study | Sample size | Age range (years) | Data source | Phenotype | Validation technique | Predictive algorithm |
|---|---|---|---|---|---|---|
| Erus et al.[23] | 621 | 8–22 | PNC | cognition | 10-fold | SVR |
| Liem et al.[24] | 2,354 + 475 | 18–85 | LIFE, NKI | cognition | 5-fold | SVR + multisource RF |
| Richard et al.[25] | 612 + 265 | 18–88 | CamCAN, StrokeMRI | cognition | 10-fold | GTB |
| Cole[2] | 17,461 | 45–80 | UK Biobank | biomedical, cognition, lifestyle | 10-fold | LASSO |
| de Lange et al.[26] | 610 + 27,157 | 60–85 | Whitehall II, UK Biobank | biomedical | repeated 10-fold | GTB |
| Engemann et al.[27] | 674 | 18–87 | CamCAN | anxiety, biomedical, cognition, depression | 10-fold | stacked ensemble ML (ridge + RF) |
| Galdi et al. (2020)[28] | 105 | 0.44–0.81 | local | preterm/term birth | repeated 5-fold | ENET |
| Hu et al. (2020)[29] | 178 | 0.08–2.33 | UNC/UMN Baby Connectome Project | – | nested CV | NN |
| Niu et al.[12] | 839 | 8–21 | PNC | anxiety and PTSD, cognition | nested CV | ridge, SVR, GPR, DNN |
| Zhang et al. (2020)[30] | 1,493 | 3–21 | PING | cognition | 10-fold | non-projective dictionary learning |
| Dadi et al.[31] | 11,175 | 40–70 | UK Biobank | cognition, neuroticism (only for one model) | nested CV | RF |
| Dunas et al.[32] | 351 | 25–85 | Betula longitudinal study | cognition, education, fitness | 10-fold | BRR, LASSO, ENET, SVR, RVR, GPR |
| Luna et al. (2021)[33] | 489 | 5–17 | HBN | cognition, general psychopathology | 5-fold | stacked ensemble ML (GTB, GLM, RF, MLP, SEML) |
| Rokicki et al.[34] | 1,244 | 18–86 | TOP, NorCog, StrokeMRI | Alzheimer’s disease, bipolar disorder, cognitive impairment, schizophrenia | 10-fold | RF |
| Xifra-Porxas et al.[35] | 613 | 18–88 | CamCAN | – | nested CV | RF |
| Chen et al.[36] | 829 | 14–92 | local community | schizophrenia | 10-fold | feedforward cascade NN, GPR |
| Chen et al.[37] | 636 + 482 | 18–88 | CamCAN, local community | biomedical, cognition | 10-fold | previously trained feedforward cascade NN |
| Huang et al. (2022)[38] | 343 | 20–60 | local community | schizophrenia | leave one out | LASSO MLR |
| Ramduny et al.[39] | 50 | 65–84 | local community | lifestyle (sleep) | repeated 5-fold | multiple regression |
| Yu et al.[40] | 196 + 91 | 60–85 | ADNI, local community | cognitive impairment, genetics | repeated 5-fold | ridge |

PTSD, post-traumatic stress disorder; CV, cross-validation. Predictive algorithms: SVR, support vector regression; RF, random forest regression; ENET, elastic net regression; GTB, gradient tree boosting regression; LASSO, least absolute shrinkage and selection operator regression; NN, neural network; GPR, Gaussian process regression, DNN, deep neural network; NPDL, non-projective dictionary learning; BRR, Bayesian ridge regression; RVR, relevance vector regression; GLM, general linear model; MLP, multilayer perceptron; SEML, stacked ensemble machine learning; OLS, ordinary least squares regression; MLR, multilinear regression. Data source: PNC, Philadelphia Neurodevelopmental Cohort; PING, Pediatric Imaging, Neurocognition, and Genetics Data Repository; LIFE, Leipzig Research Center for Civilization Diseases Adult Study; NKI, Nathan Kline Institute – Rockland Sample; CamCAN, Cambridge Center for Aging and Neuroscience; TOP, Thematically Organized Psychosis study; NorCog, Norwegian Registry for Persons Assessed for Cognitive Symptoms; ADNI, Alzheimer’s Disease Neuroimaging Initiative; HBN, Healthy Brain Network.

bias.[19] Secondly, we repeated the quantitative synthesis procedure to investigate model utility (aim 2). Subsequently, we qualitatively evaluated whether the utility of brain age models depends on the phenotype in question as well as identified which phenotypes were most strongly correlated with brain age gaps. Lastly, we illustrated the relationship between model accuracy and utility both within each study and across this body of literature (aim 3). Altogether, this review examined which specific neuroimaging features provided the largest added value for both model accuracy and utility. Such insight may prove useful for understanding how best to model age-related pathology using a brain age framework.

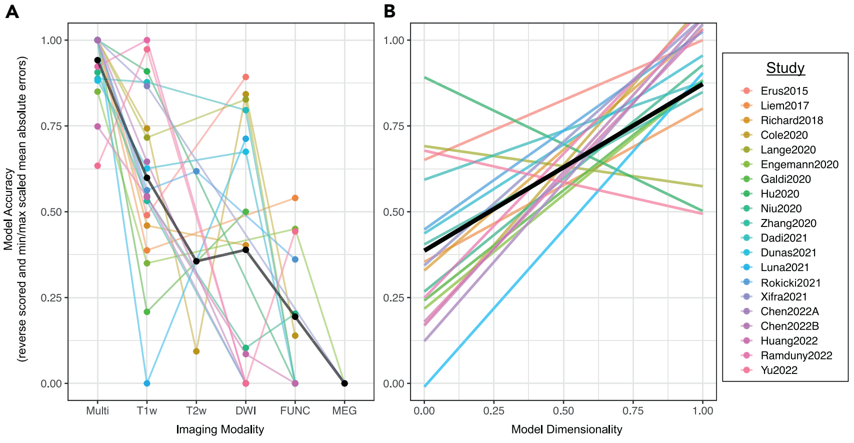


**Figure 2. The most accurate brain age models were trained with multiple modalities and the greatest number of features possible**

(A) The multimodal models were the most accurate in all but two studies. The second and third most accurate brain age models were derived exclusively from T1-weighted and DWI-weighted features, respectively.

(B) Model accuracy varied as a function of the number of features used to train each model. The three studies that were outliers in this scatterplot could be attributed to their use of highly dimensional functional connectivity features, which were not as accurate as brain morphometry or white-matter microstructure. Scaled mean absolute errors (MAEs) with a score of 1 were the most accurate within a given study, while those with a 0 were the least accurate. Each colored line represents the average of all models from a specific study, while the back line represents all models across all studies.

## METHODS

This systematic review was conducted in accordance with the Preferred Reporting Items for Systematic Reviews and Meta-analyses (PRISMA)[20] whenever possible. As such, our four-step PRISMA flow diagram is displayed in Figure 1.

### Information sources and search strategy

On May 16, 2022, literature searches were conducted across four databases (Web of Science, ScienceDirect, PubMed, and Scopus) without any limits to publication date.

We aggregated a total of 351 reports that used the term "brain age" in their title, abstract, or keywords and included the term "multimodal" anywhere throughout the article, including the references. The references for these articles were also screened yielding four additional relevant reports. After removing duplicates (n = 81) and non-empirical investigations (n = 77), we obtained 197 studies whose titles and abstracts were screened by one co-author (M.M.G.). Studies were excluded if they did not train a statistical model to predict age from neuroimaging data (n = 37) or were not conducted on human subjects (n = 6). This screening process yielded 154 studies whose full text was independently assessed for eligibility by two authors (M.M.G. and R.J.J.). Any discrepancies (n = 1) were discussed and resolved through consensus.

### Eligibility criteria

Given our interest in assessing whether multimodal features improved the accuracy and utility of brain age models, we selected studies that developed multiple models across a variety of modalities, with each model being derived from a distinct set of neuroimaging features. Studies comparing the accuracy and utility of unimodal vs. multimodal brain age models were particularly relevant for this review. Therefore, we excluded all studies that only used data from a single imaging modality (n = 81), developed only one multimodal brain age model and did not compare with any unimodal models (n = 44), or did not include at least one multimodal brain age model in their comparison of model accuracy (n = 9). Ultimately, twenty studies met our inclusion criteria, but only a subset of these studies evaluated both the accuracy and utility of their models. Specifically, ten of these studies either did not evaluate individual differences in any phenotype (n = 3) or only assessed the utility of their multimodal model without comparing its performance with their unimodal models (n = 7). Thus, our assessment of model utility was based on a subset of ten studies, and our review of model accuracy utilized all twenty studies.

### Data extraction

Information from each study was compiled and organized into three categories. First, descriptive information regarding the study design and sample characteristics was gathered (Table 1). These data included the sample size, age distribution, phenotype(s), data source(s), validation technique(s), and predictive algorithm(s). Second, the neuroimaging modalities and specific feature types were collected (Table S1). This body of literature encompassed data across multiple MRI sequences, positron emission tomography (PET), magnetoencephalography (MEG), and electroencephalography (EEG). Lastly, the relevant meta-data from each brain age model across all studies were extracted to conduct a quantitative synthesis.[18] For each brain age model, the following information was recorded: neuroimaging modality; feature type; number of input features; mean absolute error (i.e., operationalized as model accuracy); Pearson correlation between chronological and brain ages; coefficient of determination between chronological and brain ages; phenotype (i.e., cognition, psychopathology, etc.); correlation between a phenotype of interest and brain age gaps (i.e., operationalized as model utility); and reported effect size metrics (i.e., Pearson correlation, Cohen's D, etc.). This aggregated set of information is accessible via the following GitHub repository: https://zenodo.org/badge/latestdoi/549260376.[21,22]

### Data synthesis

As discussed in prior reviews of the brain age framework,[41] several confounds need to be accounted for when interpreting differences between studies. It has been demonstrated that the accuracy of brain age models varies as a function of sample size,[42] age distribution,[41] developmental stage,[43] predictive algorithm,[13] and correction methods to remove prediction biases.[44] This heterogeneity can obscure our ability to infer how the predictive power of a brain age model is related to certain imaging modalities and their corresponding neural properties. Additionally, brain age models have been trained at various scales of neuroimaging data, including voxel-level data from spatial maps,[11] extracted properties from regional atlases,[45] and higher-order covariance components derived via factor analysis methods.[31] To ensure that our results were not driven by these unwanted sources of variation, we minimum/maximum (min/max) scaled the mean absolute errors and the number of input features across all models within a given study. Additionally, we reverse scored the scaled mean absolute errors to make our results easier to interpret (i.e., being in the same direction as model utility). Therefore, the brain age models with a score of 1 were the most accurate within a given study, while those with a 0 were the least accurate. We based our assessment of model accuracy on the mean absolute errors as opposed to the coefficient of determination for two reasons: (1) it has been demonstrated that correlations are artificially weaker when the age range of the training and testing samples is restricted,[14,15] and (2) correlations between chronological and brain ages were only reported for twelve of the twenty included studies (Table S2).

### Effect measures

The studies included in this review evaluated the utility of their models across a wide range of phenotypes (e.g., cognitive functioning, mental disorder), consisting of dimensional and categorical measures. Consequently, different types of effect sizes were reported, including h-statistics,[24] Pearson correlations,[25,32,39,40] Cohen's D,[34,36] and standardized beta coefficients.[27,37,41] We standardized these indices by converting all measures of effect size to Pearson correlations and subsequently taking their absolute values. These values were used to quantify a model's utility or more specifically their capacity to detect differences for a given phenotype, regardless of the direction of the association (Table S3). If such information could not be extracted from a research article, it was computed from publicly available data[25,31] or the corresponding author was contacted via e-mail.[27,40] As a result, the relevant data were obtained across all twenty studies, thereby bypassing issues of missingness.

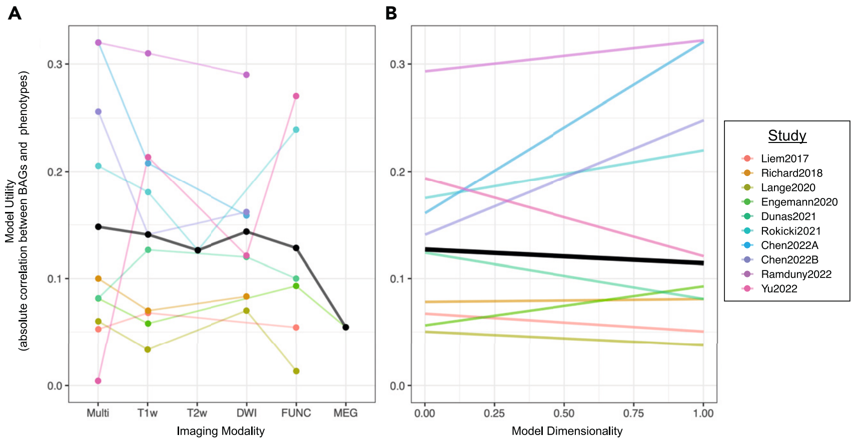


**Figure 3. The most useful brain age models varied from study to study**

(A) Model utility varied considerably across studies, such that no single set of features emerged as being substantially more useful.

(B) The utility of brain age models did not vary as a function of the number of features used to train them. Each colored line represents the average across all models from a specific study, while the back line represents all models across all studies.

### Statistical analyses

R v.4.0.2.[46] was used for analytical and visual purposes. Specifically, we used software from the following packages: *effectsize*,[47] *ggplot2*,[48] *lmer*,[49] and *lmerTest*.[50] All code and data pertaining to this review are available through the following GitHub repository: https://zenodo.org/badge/latestdoi/549260376.

Our first set of analyses were conducted across twenty brain age studies and investigated differences in the accuracy of brain age models. As such, we used mixed-effects analysis of variance to test whether model accuracy differed between imaging modalities: $anova(lmer(accuracy \sim modality + (1 \mid study)))$. Additionally, we used a mixed-effects linear model to test whether model accuracy varied as a function of the number of neuroimaging features used to train the models: $lmer(accuracy \sim dimensionality + (1 \mid study))$. Sensitivity analyses were conducted to further evaluate whether the potential association between model accuracy and dimensionality was moderated by sample size, $lmer(accuracy \sim dimensionality * sample\ size + (1 \mid study))$, or imaging modality, $lmer(accuracy \sim dimensionality* \ modality + (1 \mid study))$. For each mixed-effects model outlined above, we used a random intercept that grouped observations by the study they were obtained from.

A second set of analyses were conducted across a subset of ten studies and investigated differences in the utility of brain age models. The same analytical procedure that was used to investigate model accuracy was also used to investigate model utility, except we used a random intercept that grouped each observation by a phenotype of interest that was nested within a given study: $ANOVA(lmer(utility \sim modality + (1 \mid study/phenotype)))$ and $lmer(utility \sim dimensionality + (1 \mid study/phenotype))$. This change to the random intercept was necessary to account for the fact that some studies evaluated more than one phenotype. Lastly, a linear mixed-effects models was also used to evaluate the relationship between accuracy and utility: $lmer(accuracy \sim utility + (1 \mid study/phenotype))$.

## RESULTS

### Characteristics of included studies

Significant heterogeneity in sample characteristics and methodological choices was observed across all twenty studies that met our inclusion criteria (Table 1). Specifically, a large number of studies used open-source neuroimaging datasets, with the most popular being from the Cambridge Center for Aging and Neuroscience dataset[51] (n = 4) and the UK Biobank[52] (n = 3). Most of the studies (n = 8) were trained and tested on cross-sectional samples with age ranges spanning from late adolescence to late adulthood (18–85 years). Datasets varied considerably in size, as the smallest comprised of 50 participants, while the largest contained 17,461. Additionally, a variety of phenotypes were used to evaluate the utility of these models, with the most common phenotypes relating to cognitive function and physical fitness. Lastly, there was considerable heterogeneity with respect to validation methods and predictive algorithms.

Each study applied at least one of the following statistical models on neuroimaging data with the goal of continuously predicting age: seven used either regularized (e.g., least absolute shrinkage and selection operator),[53] Ridge,[54] ElasticNet,[55] or non-regularized (e.g., ordinary least squares) linear regression;[56] eight used support vector regression with a non-linear/radial basis kernel or tree-based algorithms (e.g., random forest);[57] gradient tree boosting;[58] five used a deep-learning framework (i.e., neural networks);[59] and three used an ensemble modeling or model stacking approach[60] (Table 1).

Each of these twenty studies used different imaging modalities and neural properties (Table S2). Briefly, all studies extracted anatomical properties derived from T1-weighted MRI scans, such as brain volume, cortical thickness, and surface area. Diffusion-weighted imaging (DWI) was the second most common modality from which indices of brain microstructure were computed using diffusion tensor imaging[61] (DTI) or neurite orientation and dispersion density imaging[62] (NODDI). The third most common imaging modality was resting-state fMRI, except for one study that also evaluated task-based MRI wherein participants engaged in an emotional face matching task.[63] Functional connectivity was the most commonly extracted feature from fMRI scans, as only two studies evaluated regional homogeneity[64] or the amplitude of low-frequency fluctuations.[65] Some additional modalities that were less common included T2-weighted MRI, arterial spin-labeling MRI, and MEG. Nine out of these twenty studies compared brain features from three or more imaging modalities.

### Aim 1: Multimodal imaging improves the accuracy of brain age models

Collectively, 117 models were developed across all twenty brain age studies, with each model being derived from a distinct set of neuroimaging data. Across this body of literature, the multimodal models were the most accurate (Figure 2A; Table S2) compared with all other groups of models that were derived from a single imaging source ($\beta > 0.24$, $p < 0.002$). This converging finding was supported by analyzing differences within each study, as the multimodal models were most accurate in all but two cases; even so, in those two atypical cases, the multimodal models were only slightly less accurate than the anatomical models derived solely from T1-weighted scans.[39,40] Further, training brain age models with the greatest number of features yielded the lowest prediction errors ($\beta = 0.52$, $p < 0.001$; Figure 2B), and this association was not moderated by sample size or imaging modality

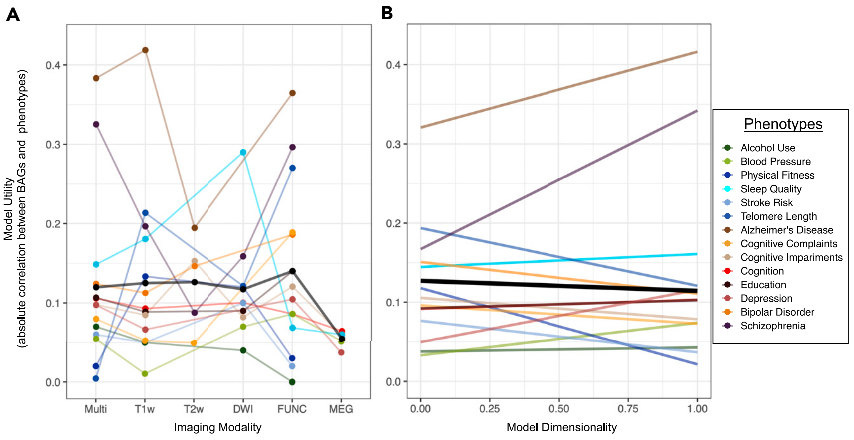


**Figure 4. The utility of brain age models differed between phenotype of interest**

(A) The fMRI brain age models exhibited a slight advantage in utility. However, multimodal models yielded the strongest effect sizes when detecting differences in chronic brain disorders, including Alzheimer's disease and schizophrenia.

(B) Similarly, brain age models that were derived with a greater number of features were generally better at detecting differences in chronic brain disorders. Each colored line represents the average across all models from a specific study, while the back line represents all models across all studies.

(Figure S2). In cases when less accurate or "noisy" features were included, the accuracy of brain age models did not decline.[26] However, adding more features from a single modality did not benefit model accuracy as much as adding complimentary information from another modality.[24] Taken together, these results indicate that both incorporating multiple modalities and, to a lesser extent, increasing the number of features were essential for improving a model's capacity to accurately predict age.

Although the multimodal brain age models were most accurate, certain modalities had a disproportionate impact on the predictive power of brain age models. Specifically, models trained on anatomical features (i.e., cortical thickness and volume) or white matter microstructure (i.e., mean diffusivity and apparent diffusion coefficient) were consistently more accurate than those derived from markers of brain activation (i.e., amplitude of low frequency fluctuations) or functional organization (i.e., functional connectivity; Figure 2A). Further, most of the studies included in this review (n = 12) performed feature importance analyses to illustrate which individual regions had a significant contribution to brain age predictions. Across these analyses, the features that consistently had the largest importance were morphometric measurements of the precuneus, orbitofrontal cortex, and deep brain structures. Specifically, the predictive power of brain age models could be most attributed to the thalamus, striatum, cingulate, insula, ventricles, hippocampus, caudate, and amygdala.[12,35] Additionally, the white matter tracts that contributed the most to the predictive power of brain age models included the corpus callosum, choroid plexus, fornix, and uncinate fasciculus.[2,26,34] Therefore, the most accurate unimodal brain age models appeared to be those that were based on anatomical, as opposed to functional, MRI properties.

Another dimension of model accuracy is age-related prediction bias, which is a well-known limitation of the brain age framework.[19] This bias refers to the fact that brain ages of younger individuals are overestimated while the predictions of older individuals are underestimated.[44] Nearly all unimodal models are universally affected by this bias,[12] but it is unclear whether multimodal models are also susceptible. Through our review of the multimodal literature, we identified only three models whose brain age predictions were not systematically biased, as their brain age gaps did not vary as a function of age.[2,35,66] A commonality between these three models was that they all applied traditional machine-learning models to spatial maps from structural MRI sequences, primarily T1-weighted and diffusion-weighted scans. However, other studies used similar approaches and obtained models whose predictions were still susceptible to biases.[23,32] Therefore, it is still not robustly clear whether using multimodal imaging or certain feature types can mitigate issues of prediction bias.

## Aim 2: Model utility depends on both imaging modality and the phenotype of interest

Among the ten studies that compared the utility of multiple brain age models, a total of 55 models were developed and evaluated using 13 distinct phenotypes (Figure S3). Similar to our assessment of model accuracy, the multimodal brain age models were the most useful across all studies, but such differences were only statistically significant when compared with the models trained with features derived solely from T2-weighted or MEG scans ($\beta > 0.22$, $p < 0.002$; Figure 3A). Further, the utility of all brain age models did not differ by the number of neuroimaging features upon which they were trained ($\beta = 0.02$, $p = 0.75$; Figure 3B). It is possible that significant differences in model utility did not emerge (relative to model accuracy) because of the heterogeneity in phenotypes of interest and discrepancies in the methods used to quantify them.

To understand whether the utility of brain age models depended on the phenotype in question, we narrowed our focus on two studies that contained the largest number of phenotypes. Rokicki et al.[34] demonstrated that nearly all brain age models were able to detect differences in Alzheimer's disease and schizophrenia. However, brain age models that were derived from either multimodal features or strictly morphometric features from T1-weighted MRI scans yielded the largest effect sizes. In contrast, models that were based on cerebral blood flow from arterial spin-labeling MRI scans were the only ones to be significantly associated with all five of their phenotypes, including bipolar disorder, cognitive complaints, and mild cognitive impairments. These findings partially aligned with Engemann et al.,[27] who reported that multimodal models yielded the strongest effect sizes, whereas models derived exclusively from brain activation (via MEG) and functional connectivity (via fMRI) were related to certain phenotypes that other models could not detect. Taken together,

CellPress
OPEN ACCESS

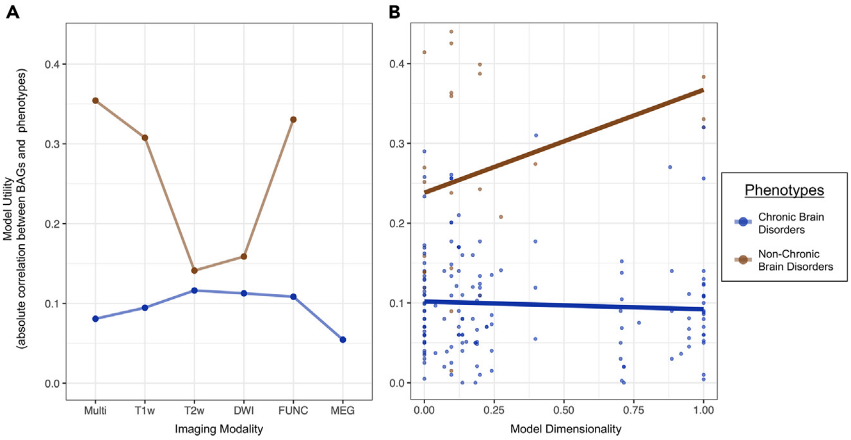


**Figure 5. Brain age models were best at detecting differences in chronic brain disorders**

(A) Brain age models were best at detecting differences in Alzheimer's disease and schizophrenia. These improvements in model utility were most prominent when using multimodal, anatomical, or functional MRI features.

(B) As the number of features increased, brain age models were better at detecting differences in these chronic brain conditions, but this was not the case when grouping together all twelve of the remaining phenotypes.

models derived from multimodal or morphometric brain features appear to yield the strongest correlations among a limited number of phenotypes, whereas those derived from functional imaging modalities were sensitive to the largest number of phenotypes.

To further investigate whether the utility of brain age models depends on the phenotype in question, we assessed these relationships across all ten studies using a quantitative synthesis approach.[18] As expected, Alzheimer's disease and schizophrenia exhibited the strongest effect sizes (Figure 4A), especially for the multimodal, T1-weighted, or functional brain age models. In contrast, all other phenotypes exhibited weaker associations that were best detected by the functional brain age models. These dynamics were also reflected in the relationship between model utility and the number of features used to train each model (Figure 4B). Specifically, brain age models that used the largest number of features were more able to detect differences in Alzheimer's disease and schizophrenia, but these relationships were stable or decreasing for almost all other phenotypes. Therefore, it appears that model utility depends on both the phenotype of interest and neuroimaging features used (Figure 5), but not all studies followed this emerging pattern. In particular, Lange et al.[26] reported that models based on functional connectivity were the least useful at detecting differences in alcohol use, blood pressure, and stroke risk. It is worth noting that this study was conducted on one of the oldest cohorts, spanning from 60 to 85 years of age, which may partly explain why their results were relatively idiosyncratic.

## Aim 3: The accuracy and utility of brain age models are not related

Our review suggests that multimodal brain age models are almost always the most accurate but are not necessarily the most sensitive to differences across a variety of phenotypes. Such finding aligns with a developing narrative in the brain age literature that the accuracy and utility of brain age models are two unrelated performance metrics.[16,17] Mixed-effects models were used to further evaluate this notion across all brain age models from the ten included studies in this review. As expected, the utility of these models did not vary as a function of their accuracy ($\beta = -0.02$, $p = 0.73$; Figure 6). This result was further supported when evaluating each study individually, as the data from one study suggested that model utility was better when accuracy was worse ($\beta = -0.54$, $p = 0.04$),[26] and all other studies did not display any substantial trends or associations ($\beta < -0.33$, $p > 0.07$).

## DISCUSSION

This systematic review demonstrated that feature selection has a large downstream impact on both the accuracy and utility of brain age models. Yet, we could not identify a single set of neuroimaging features that led to uniform improvements across all performance metrics and conditions, indicating that unimodal and multimodal models each have distinct use cases. Indeed, these findings have been echoed in prior studies. Liem et al.[24] was one of the first studies to conclude that multimodal models were best at predicting age, while specific imaging modalities were superior at detecting differences in pathology. Despite mounting evidence, there is a persisting assumption across several studies that the most accurate brain age models will have the most potential for detecting differences in a given phenotype of interest. As a point of illustration, seven of the twenty studies in this review only evaluated the utility of their most accurate model, which in all cases was trained using multimodal features. This approach has also led to researchers to exclusively use T1-weighted and diffusion-weighted MRI scans when developing brain age models[36] since such modalities have been shown to have the largest contribution to a model's predictive power.[2,67] However, our review suggests that model accuracy does not necessarily provide meaningful insight about clinical utility (e.g., detection of age-related pathology). Taken with prior studies,[16,17] it appears that the most accurate models tend to not be the most useful. Yet, it is not robustly clear how accurate brain age predictions should be to increase a model's capacity for detecting individual differences.[68] These results highlight that a more exhaustive approach may be necessary when making comparisons between brain age models. For instance, evaluations of brain age models should go beyond comparing mean absolute errors and also evaluate their models' utility across a variety of phenotypes. Given that our results regarding model utility were relatively more variable across studies and based on a limited number of observations (Figure S4), we believe that further evaluating the clinical applications of unimodal vs. multimodal models remains a top priority.

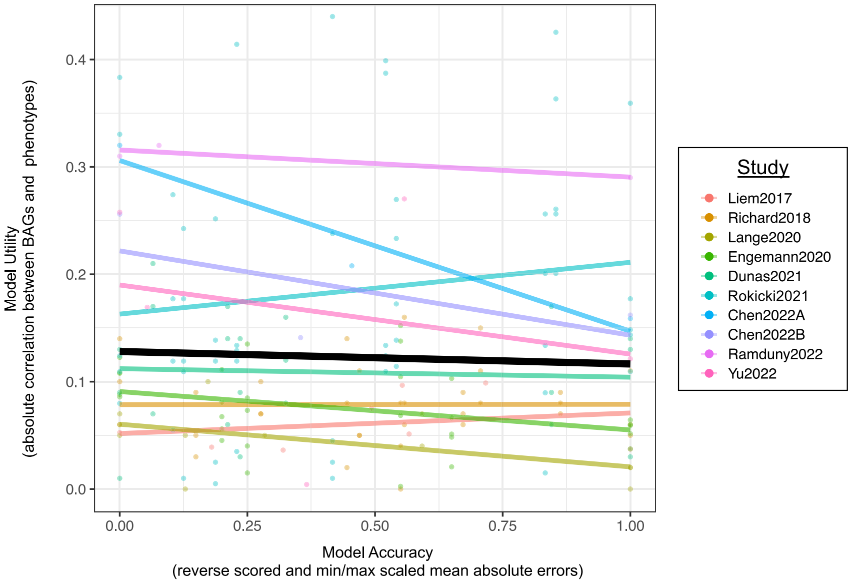


**Figure 6. The utility of brain age models did not vary as a function of their accuracy**
Each point represents the accuracy and utility of a given model, which is color coded by the study it was gathered from. The black line denotes the relationship between accuracy and utility across all models from all ten studies. Scaled MAEs with a score of 1 were the most accurate within a given study, while those with a 0 were the least accurate.

Additionally, we observed that the utility of brain age models largely depended on the phenotype of interest. In particular, brain age models were best able to explain differences in chronic brain disorders, including Alzheimer's disease and schizophrenia.[34] We propose three potential reasons as to why these specific phenotypes yielded the strongest correlations with brain age gaps. First, Alzheimer's disease and schizophrenia are progressive in nature, as the onset and severity of symptoms are robustly associated with age.[69,70] Secondly, both forms of pathology disrupt multiple facets of cognition and mental health, which may be easier to quantify or diagnose.[71] Lastly, Alzheimer's disease and schizophrenia have been shown to have severe and widespread impacts on the brain,[72,73] potentially making brain age prediction more sensitive to disruptions associated with these two illnesses. Further, the widespread brain atrophy associated with each of these illnesses is reflected by a considerable shrinkage in brain volume,[74] which is a feature type that is heavily weighted by multimodal brain age models. Altogether, these factors may each contribute to patients with Alzheimer's disease and schizophrenia having larger brain age gaps that widen overtime.[42,75] Nevertheless, it could be beneficial for additional studies to disentangle which of these three possibilities most substantially improved the utility of brain age models.

Although Alzheimer's disease and schizophrenia were best captured by multimodal brain age models, it appeared that these models were not as useful in detecting differences in other phenotypes. Our interpretation of these results is that multimodal brain age models most heavily weigh the features that can accurately predict age, regardless of their clinical relevance. As a consequence, some features with little clinical significance still had an outsized contribution to brain age predictions, including the corpus callosum, choroid plexus, lateral ventricles, and cerebellum.[76] In contrast, numerous subcortical structures of the limbic system were also heavily weighted by brain age models, including the thalamus, hippocampus, and amygdala.[12,35] Each of these structures plays a crucial role in facilitating cognitive functioning[77] and emotional regulation.[78] Further, abnormalities in the morphometric measurements of hippocampus and amygdala are robust biomarkers of both Alzheimer's disease[72] and schizophrenia.[43] These findings may help to decode why multimodal models were sensitive to these chronic brain disorders, but ways to systematically improve their utility to detect other phenotypes remain unclear.

Aside from chronic brain disorders, all other phenotypes were robustly associated with brain age models that were derived solely from fMRI features. Nearly all the included studies reported that features of brain activation or functional organization encoded unique information that was useful in detecting individual differences.[27,34] However, when functional features were combined with metrics of brain morphometry or white-matter microstructures, we observed a reduction in model utility. This consequence might have occurred because multimodal brain age models more heavily prioritize structural features due to their robust associations with age and reliability across the lifespan.[35] Although functional brain features have a limited capacity to accurately predict age, they appear to be more sensitive to transitory life events or state-like phenotypes.[26] It is possible that fMRI features were more sensitive to earlier indicators of pathology because they are more malleable and likely to precede changes in brain structure.[79] Taken further, these precedents suggest that fMRI features could be more useful in predicting pathology directly. Future longitudinal studies are needed to determine how structure and function dynamics across time may lead to differences in the utility of these machine-learning frameworks. Altogether, fMRI features appear to have the most potential for developing brain age models with widespread utility.

This systematic review contains several limitations. Accounting for all unwanted sources of variation when making inferences between brain age studies can be challenging because of differences in sample characteristics and methodological decisions.[41] We addressed these challenges by min/max scaling the mean absolute errors and the number of features used for each model from a given study, thereby placing emphasis on within- and across-study differences. Therefore, our conclusions that are based on these standardized metrics may be confounded by differences between studies, such as sample demographics, clinical phenotypes, social factors, data quality, model complexity, and regularization techniques. For example, differences in regularization techniques can largely influence the accuracy and utility of brain age models

as well as the stability of the model weights.[15] Further, our quantitative synthesis was limited in statistical power, especially when evaluating the utility for a given set of neuroimaging features by specific phenotypes of interest. At times, we only had access to the results of a single study, such as those from Rokicki et al.,[34] which was the only multimodal study to evaluate Alzheimer's disease, bipolar disorders, and cognitive complaints. Given these limits in statistical power, it was not feasible to employ formal meta-analysis methods like those used for quantifying the heterogeneity between studies.[80] Nevertheless, it was encouraging that our findings overlapped with unimodal brain age studies that were not included in this review, but also demonstrated that brain age gaps were most sensitive to differences in chronic brain disorders, including Alzheimer's disease and schizophrenia.[6,42]

Since the first multimodal brain age study was published in 2011, the enthusiasm for using multimodal imaging has only increased. Theoretically, providing brain age models with complimentary information about brain development should result in more nuanced predictions that can better reflect individual differences. In practice, the primary advantage of multimodal models was an increased capacity to predict age, which did not translate to widespread improvements in their utility. These conclusions build upon prior demonstrations that the most accurate models tend to not be the most useful.[16,17] Additionally, it could be argued that providing brain age models with a larger feature space makes them more susceptible to issues of overfitting.[81] Taken together, we believe that more creative approaches are needed to further maximize the benefits that brain age models could leverage from multimodal imaging. One possible avenue could be to apply a fitness function to a set of brain age gaps with the goal of maximizing the separability between individuals based on a selected distance criterion. This approach has been shown to improve sensitivity to Alzheimer's disease[82] and could have greater implications if applied to multimodal models. However, researchers must consider whether the added benefits of multimodal brain age models outweigh the costs of limited transferability.[83–85] Specifically, more data will need to be collected for a multimodal model to be applied, which is not always feasible, especially in clinical settings. Further, more work is needed to evaluate higher-order features of brain networks. For example, the edge strength was the most commonly used fMRI feature across this body of literature. It is unknown whether indices of graph theory[86] or network cohesion[87] could lead to even greater improvements in model utility. To conclude, multimodal imaging has provided us with valuable insight for improving the accuracy of brain age models, but there is still much untapped potential in regard to achieving widespread clinical utility.

## SUPPLEMENTAL INFORMATION

Supplemental information can be found online at https://doi.org/10.1016/j.patter.2023.100712.

## ACKNOWLEDGMENTS

We would like to thank Tom Earnest, who helped us with identifying and screening empirical studies. We would also like to acknowledge Yu Junhong, who helped us obtain the relevant metadata from their previously published study, which enabled us to perform our quantitative synthesis without issues of missingness. This work was supported by the National Science Foundation Graduate Research Fellowship (DGE-2139839 and DGE-1745038).

## AUTHOR CONTRIBUTIONS

Conceptualization, R.J.J., A.J.G., R.B., D.M.B., and A.S.; methodology, R.J.J, D.M.B., and A.S.; validation, R.J.J. and M.M.G.; curation, R.J.J., M.M.G., and D.A.E.; formal analysis, R.J.J.; writing – original draft, R.J.J., A.J.G., and M.M.G.; writing – review & editing, R.J.J., D.A.E., R.B., D.M.B., and A.S.; visualization, R.J.J.; supervision, D.M.B. and A.S.

## DECLARATION OF INTERESTS

D.A.E. is a full-time employee of F. Hoffmann-La Roche, Ltd. A.S. is a shareholder in TheraPanacea and a paid consultant for the BrightFocus Foundation.

## INCLUSION AND DIVERSITY

We support inclusive, diverse, and equitable conduct of research.

## REFERENCES

1. Singh, N.M., Harrod, J.B., Subramanian, S., Robinson, M., Chang, K., Cetin-Karayumak, S., Dalca, A.V., Eickhoff, S., Fox, M., Franke, L., et al. (2022). How machine learning is powering neuroimaging to improve brain health. Neuroinformatics *20*, 943–964. https://doi.org/10.1007/s12021-022-09572-9.

2. Cole, J.H. (2020). Multimodality neuroimaging brain-age in UK biobank: relationship to biomedical, lifestyle, and cognitive factors. Neurobiol. Aging *92*, 34–42. https://doi.org/10.1016/j.neurobiolaging.2020.03.014.

3. Elliott, M.L., Belsky, D.W., Knodt, A.R., Ireland, D., Melzer, T.R., Poulton, R., Ramrakha, S., Caspi, A., Moffitt, T.E., and Hariri, A.R. (2021). Brain-age in midlife is associated with accelerated biological aging and cognitive decline in a longitudinal birth cohort. Mol. Psychiatr. *26*, 3829–3838. https://doi.org/10.1038/s41380-019-0626-7.

4. Jawinski, P., Markett, S., Drewelies, J., Düzel, S., Demuth, I., Steinhagen-Thiessen, E., Wagner, G.G., Gerstorf, D., Lindenberger, U., Gaser, C., and Kühn, S. (2022). Linking brain age gap to mental and physical health in the berlin aging study II. Front. Aging Neurosci. *14*, 791222. https://doi.org/10.1126/science.aac4716.

5. Ballester, P.L., Romano, M.T., de Azevedo Cardoso, T., Hassel, S., Strother, S.C., Kennedy, S.H., and Frey, B.N. (2022). Brain age in mood and psychotic disorders: a systematic review and meta-analysis. Acta Psychiatr. Scand. *145*, 42–55. https://doi.org/10.1111/acps.13371.

6. Millar, P.R., Luckett, P.H., Gordon, B.A., Benzinger, T.L.S., Schindler, S.E., Fagan, A.M., Cruchaga, C., Bateman, R.J., Allegri, R., Jucker, M., et al. (2022). Predicting brain age from functional connectivity in symptomatic and preclinical Alzheimer disease. Neuroimage *256*, 119228. https://doi.org/10.1016/j.neuroimage.2022.119228.

7. Brown, T.T., Kuperman, J.M., Chung, Y., Erhart, M., McCabe, C., Hagler, D.J., Venkatraman, V.K., Akshoomoff, N., Amaral, D.G., Bloss, C.S., et al. (2012). Neuroanatomical assessment of biological maturity. Curr. Biol. *22*, 1693–1698. https://doi.org/10.1016/j.cub.2012.07.002.

8. Landhuis, E. (2017). Neuroscience: big brain, big data. Nature *541*, 559–561. https://doi.org/10.1038/541559a.

9. Barch, D.M. (2018). The power of collaboration and data aggregation. Biol. Psychiatr. *84*, 626–628. https://doi.org/10.1016/j.biopsych.2018.06.005.

10. Pomponio, R., Erus, G., Habes, M., Doshi, J., Srinivasan, D., Mamourian, E., Bashyam, V., Nasrallah, I.M., Satterthwaite, T.D., Fan, Y., et al. (2020). Harmonization of large MRI datasets for the analysis of brain imaging patterns throughout the lifespan. Neuroimage *208*, 116450. https://doi.org/10.1016/j.neuroimage.2019.116450.

11. Baecker, L., Garcia-Dias, R., Vieira, S., Scarpazza, C., and Mechelli, A. (2021). Machine learning for brain age prediction: introduction to methods and clinical applications. EBioMedicine *72*, 103600. https://doi.org/10.1016/j.ebiom.2021.103600.

12. Niu, X., Zhang, F., Kounios, J., and Liang, H. (2020). Improved prediction of brain age using multimodal neuroimaging data. Hum. Brain Mapp. *41*, 1626–1643. https://doi.org/10.1002/hbm.24899.

13. Schulz, M.-A., Yeo, B.T.T., Vogelstein, J.T., Mourao-Miranada, J., Kather, J.N., Kording, K., Richards, B., and Bzdok, D. (2020). Different scaling of linear models and deep learning in UKBiobank brain images versus machine-learning datasets. Nat. Commun. *11*, 4238. https://doi.org/10.1038/s41467-020-18037-z.

14. Poldrack, R.A., Huckins, G., and Varoquaux, G. (2020). Establishment of best practices for evidence for prediction: a review. JAMA Psychiatr. *77*, 534–540. https://doi.org/10.1001/jamapsychiatry.2019.3671.

15. Varoquaux, G., Raamana, P.R., Engemann, D.A., Hoyos-Idrobo, A., Schwartz, Y., and Thirion, B. (2017). Assessing and tuning brain decoders: cross-validation, caveats, and guidelines. Neuroimage *145*, 166–179. https://doi.org/10.1016/j.neuroimage.2016.10.038.

16. Bashyam, V.M., Erus, G., Doshi, J., Habes, M., Nasrallah, I., Truelove-Hill, M., Srinivasan, D., Mamourian, L., Pomponio, R., Fan, Y., et al. (2020). MRI signatures of brain age and disease over the lifespan based on a deep brain network and 14 468 individuals worldwide. Brain *143*, 2312–2324. https://doi.org/10.1093/brain/awaa160.

17. Jirsaraie, R.J., Kaufmann, T., Bashyam, V., Erus, G., Luby, J.L., Westlye, L.T., Davatzikos, C., Barch, D.M., and Sotiras, A. (2022). Benchmarking the generalizability of brain age models: challenges posed by scanner variance and prediction bias. Hum. Brain Mapp. *44*, 1118–1128. https://doi.org/10.1002/hbm.26144.

18. Lau, J., Ioannidis, J.P., and Schmid, C.H. (1997). Quantitative synthesis in systematic reviews. Ann. Intern. Med. *127*, 820–826. https://doi.org/10.7326/0003-4819-127-9-199711010-00008.

19. Smith, S.M., Vidaurre, D., Alfaro-Almagro, F., Nichols, T.E., and Miller, K.L. (2019). Estimation of brain age delta from brain imaging. Neuroimage *200*, 528–539. https://doi.org/10.1016/j.neuroimage.2019.06.017.

20. Page, M.J., McKenzie, J.E., Bossuyt, P.M., Boutron, I., Hoffmann, T.C., Mulrow, C.D., Shamseer, L., Tetzlaff, J.M., Akl, E.A., Brennan, S.E., et al. (2021). The PRISMA 2020 statement: an updated guideline for reporting systematic reviews. Syst. Rev. *372*, n71. https://doi.org/10.1136/bmj.n71.

21. Smyth, V. (2021). How to Cite and Reference in Harvard Style (v.1_130621) (Zenodo). https://doi.org/10.5281/zenodo.4940014.

22. RobertJirsaraie; CCP Lab @ WUSTL (2022). RobertJirsaraie/Robert-Jirsaraie: Completion of the Proj21-DivergeReview (Proj21-DivergeReview) (Zenodo). https://doi.org/10.5281/zenodo.7183682.

23. Erus, G., Battapady, H., Satterthwaite, T.D., Hakonarson, H., Gur, R.E., Davatzikos, C., and Gur, R.C. (2015). Imaging patterns of brain development and their relationship to cognition. Cerebr. Cortex *25*, 1676–1684. https://doi.org/10.1093/cercor/bht425.

24. Liem, F., Varoquaux, G., Kynast, J., Beyer, F., Kharabian Masouleh, S., Huntenburg, J.M., Lampe, L., Rahim, M., Abraham, A., Craddock, R.C., et al. (2017). Predicting brain-age from multimodal imaging data captures cognitive impairment. Neuroimage *148*, 179–188. https://doi.org/10.1016/j.neuroimage.2016.11.005.

25. Richard, G., Kolskår, K., Sanders, A.M., Kaufmann, T., Petersen, A., Doan, N.T., Monereo Sánchez, J., Alnæs, D., Ulrichsen, K.M., Dørum, E.S., et al. (2018). Assessing distinct patterns of cognitive aging using tissue-specific brain age prediction based on diffusion tensor imaging and brain morphometry. PeerJ *6*, e5908. https://doi.org/10.7717/peerj.5908.

26. de Lange, A.M.G., Anatürk, M., Suri, S., Kaufmann, T., Cole, J.H., Griffanti, L., Zsoldos, E., Jensen, D.E.A., Filippini, N., Singh-Manoux, A., et al. (2020). Multimodal brain-age prediction and cardiovascular risk: the Whitehall II MRI sub-study. Neuroimage *222*, 117292. https://doi.org/10.1016/j.neuroimage.2020.117292.

27. Engemann, D.A., Kozynets, O., Sabbagh, D., Lemaître, G., Varoquaux, G., Liem, F., and Gramfort, A. (2020). Combining magnetoencephalography with magnetic resonance imaging enhances learning of surrogate-biomarkers. Elife *9*, e54055. https://doi.org/10.7554/eLife.54055.

28. Galdi, P., Blesa, M., Stoye, D.Q., Sullivan, G., Lamb, G.J., Quigley, A.J., Thrippleton, M.J., Bastin, M.E., and Boardman, J.P. (2020). Neonatal morphometric similarity mapping for predicting brain age and characterizing neuroanatomic variation associated with preterm birth. NeuroImage: Clinical *25*. https://doi.org/10.1016/j.nicl.2020.102195.

29. Hu, D., Zhang, H., Wu, Z.W., Wang, F., Wang, L., Smith, J.K., Lin, W.L., Li, G., and Shen, D.G. (2020). Disentangled-Multimodal Adversarial Autoencoder: Application to Infant Age Prediction With Incomplete Multimodal Neuroimages. IEEE Trans. Med. Imaging *39*, 4137–4149. https://doi.org/10.1109/TMI.2020.3013825.

30. Zhang, M., Desrosiers, C., Guo, Y., Khundrakpam, B., Al-Sharif, N., Kiar, G., Valdes-Sosa, P., Poline, J.B., and Evans, A. (2020). Brain status modeling with non-negative projective dictionary learning. NeuroImage *206*. https://doi.org/10.1016/j.neuroimage.2019.116226.

31. Dadi, K., Varoquaux, G., Houenou, J., Bzdok, D., Thirion, B., and Engemann, D. (2021). Population modeling with machine learning can enhance measures of mental health. GigaScience *10*, giab071. https://doi.org/10.1093/gigascience/giab071.

32. Dunås, T., Wåhlin, A., Nyberg, L., and Boraxbekk, C.J. (2021). Multimodal image analysis of apparent brain age identifies physical fitness as predictor of brain maintenance. Cereb. Cortex *31*, 3393–3407. https://doi.org/10.1093/cercor/bhab019.

33. Luna, A., Bernanke, J., Kim, K., Aw, N., Dworkin, J.D., Cha, J., and Posner, J. (2021). Maturity of gray matter structures and white matter connectomes, and their relationship with psychiatric symptoms in youth. Hum. Brain Mapp. *42*, 4568–4579. https://doi.org/10.1002/hbm.25565.

34. Rokicki, J., Wolfers, T., Nordhøy, W., Tesli, N., Quintana, D.S., Alnaes, D., Richard, G., de Lange, A.-M.G., Lund, M.J., Norbom, L., et al. (2021). Multimodal imaging improves brain age prediction and reveals distinct abnormalities in patients with psychiatric and neurological disorders. Hum. Brain Mapp. *42*, 1714–1726. https://doi.org/10.1002/hbm.25323.

35. Xifra-Porxas, A., Ghosh, A., Mitsis, G.D., and Boudrias, M.-H. (2021). Estimating brain age from structural MRI and MEG data: insights from dimensionality reduction techniques. Neuroimage *231*, 117822. https://doi.org/10.1016/j.neuroimage.2021.117822.

36. Chen, C.-L., Hwang, T.J., Tung, Y.-H., Yang, L.-Y., Hsu, Y.-C., Liu, C.M., Lin, Y.-T., Hsieh, M.-H., Liu, C.-C., Chien, Y.-L., et al. (2022). Detection of advanced brain aging in schizophrenia and its structural underpinning by using normative brain age metrics. Neuroimage. Clin. *34*, 103003. https://doi.org/10.1016/j.nicl.2022.103003.

37. Chen, C.-L., Kuo, M.-C., Chen, P.-Y., Tung, Y.-H., Hsu, Y.-C., Huang, C.-W.C., Chan, W.P., and Tseng, W.-Y.I. (2022). Validation of neuroimaging-based brain age gap as a mediator between modifiable risk factors and cognition. Neurobiol. Aging *114*, 61–72. https://doi.org/10.1016/j.neurobiolaging.2022.03.006.

38. Huang, J., Ke, P., Chen, X., Li, S., Zhou, J., Xiong, D., Huang, Y., Li, H., Ning, Y., Duan, X., et al. (2022). Multimodal Magnetic Resonance Imaging Reveals Aberrant Brain Age Trajectory During Youth in Schizophrenia Patients. Front. Aging Neurosci. *14*. https://doi.org/10.3389/fnagi.2022.823502.

39. Ramduny, J., Bastiani, M., Huedepohl, R., Sotiropoulos, S.N., and Chechlacz, M. (2022). The association between inadequate sleep and accelerated brain ageing. Neurobiol. Aging *114*, 1–14. https://doi.org/10.1016/j.neurobiolaging.2022.02.005.

40. Yu, J., Mathi Kanchi, M., Rawtaer, I., Feng, L., Kumar, A.P., Kua, E.-H., and Mahendran, R.; Alzheimer's Disease Neuroimaging Initiative (2022). Differences between multimodal brain-age and chronological-age are linked to telomere shortening. Neurobiol. Aging *115*, 60–69. https://doi.org/10.1016/j.neurobiolaging.2022.03.015.

41. de Lange, A.-M.G., Anatürk, M., Rokicki, J., Han, L.K.M., Franke, K., Alnæs, D., Ebmeier, K.P., Draganski, B., Kaufmann, T., Westlye, L.T., et al. (2022). Mind the gap: performance metric evaluation in brain-age prediction. Hum. Brain Mapp. *43*, 3113–3129. https://doi.org/10.1002/hbm.25837.

42. Kaufmann, T., van der Meer, D., Doan, N.T., Schwarz, E., Lund, M.J., Agartz, I., Alnæs, D., Barch, D.M., Baur-Streubel, R., Bertolino, A., et al. (2019). Common brain disorders are associated with heritable patterns

of apparent aging of the brain. Nat. Neurosci. *22*, 1617–1623. https://doi.org/10.1038/s41593-019-0471-7.

43. Amoroso, N., La Rocca, M., Bellantuono, L., Diacono, D., Fanizzi, A., Lella, E., Lombardi, A., Maggipinto, T., Monaco, A., Tangaro, S., and Bellotti, R. (2019). Deep learning and multiplex networks for accurate modeling of brain age. Front. Aging Neurosci. *11*, 115. https://doi.org/10.3389/fnagi.2019.00115.

44. Butler, E.R., Chen, A., Ramadan, R., Le, T.T., Ruparel, K., Moore, T.M., Satterthwaite, T.D., Zhang, F., Shou, H., Gur, R.C., et al. (2021). Pitfalls in brain age analyses. Hum. Brain Mapp. *42*, 4092–4101. https://doi.org/10.1002/hbm.25533.

45. Mouches, P., Wilms, M., Rajashekar, D., Langner, S., and Forkert, N.D. (2022). Multimodal biological brain age prediction using magnetic resonance imaging and angiography with the identification of predictive regions. Hum. Brain Mapp. *43*, 2554–2566. https://doi.org/10.1002/hbm.25805.

46. Team, R.C. (2013). R: A Language and Environment for Statistical Computing.

47. Ben-Shachar, M., Lüdecke, D., and Makowski, D. (2020). effectsize: estimation of effect size indices and standardized parameters. J. Open Source Softw. *5*, 2815.

48. Wickham, H., Chang, W., and Wickham, M.H. (2016). Package 'ggplot2.'. Create elegant data visualisations using the grammar of graphics. Version *2*, 1–189.

49. Bates, D. (2005). Fitting linear mixed models in R. R news *5*, 27–30.

50. Kuznetsova, A., Brockhoff, P.B., and Christensen, R.H.B. (2017). lmerTest package: tests in linear mixed effects models. J. Stat. Softw. *82*, 1–26.

51. Shafto, M.A., Tyler, L.K., Dixon, M., Taylor, J.R., Rowe, J.B., Cusack, R., Calder, A.J., Marslen-Wilson, W.D., Duncan, J., Dalgleish, T., et al. (2014). The Cambridge Centre for Ageing and Neuroscience (Cam-CAN) study protocol: a cross-sectional, lifespan, multidisciplinary examination of healthy cognitive ageing. BMC Neurol. *14*, 204.

52. Sudlow, C., Gallacher, J., Allen, N., Beral, V., Burton, P., Danesh, J., Downey, P., Elliott, P., Green, J., Landray, M., et al. (2015). UK biobank: an open access resource for identifying the causes of a wide range of complex diseases of middle and old age. PLoS Med. *12*, e1001779.

53. Kukreja, S.L., Löfberg, J., and Brenner, M.J. (2006). A least absolute shrinkage and selection operator (LASSO) for nonlinear system identification. IFAC Proc. Vol. *39*, 814–819.

54. McDonald, G.C. (2009). Ridge regression. WIREs. Comp. Stat. *1*, 93–100.

55. Zou, H., and Hastie, T. (2005). Regularization and variable selection via the elastic net. J. Royal Statistical Soc. B *67*, 301–320. https://doi.org/10.1111/j.1467-9868.2005.00503.x.

56. Zdaniuk, B. (2014). Ordinary least-squares (OLS) model. In Encyclopedia of Quality of Life and Well-Being Research, A.C. Michalos, ed. (Springer Netherlands), pp. 4515–4517. https://doi.org/10.1007/978-94-007-0753-5_2008.

57. Ho, T.K. (1995). Random decision forests. In IEEE, pp. 278–282.

58. Friedman, J.H. (2002). Stochastic gradient boosting. Comput. Stat. Data Anal. *38*, 367–378.

59. LeCun, Y., Bengio, Y., and Hinton, G. (2015). Deep learning. Nature *521*, 436–444. https://doi.org/10.1038/nature14539.

60. Wolpert, D.H. (1992). Stacked generalization. Neural Network. *5*, 241–259.

61. Le Bihan, D., Mangin, J.-F., Poupon, C., Clark, C.A., Pappata, S., Molko, N., and Chabriat, H. (2001). Diffusion tensor imaging: concepts and applications. J. Magn. Reson. Imag. *13*, 534–546. https://doi.org/10.1002/jmri.1076.

62. Zhang, H., Schneider, T., Wheeler-Kingshott, C.A., and Alexander, D.C. (2012). NODDI: practical in vivo neurite orientation dispersion and density imaging of the human brain. Neuroimage *61*, 1000–1016. https://doi.org/10.1016/j.neuroimage.2012.03.072.

63. Hariri, A.R., Tessitore, A., Mattay, V.S., Fera, F., and Weinberger, D.R. (2002). The amygdala response to emotional stimuli: a comparison of faces and scenes. Neuroimage *17*, 317–323. https://doi.org/10.1006/nimg.2002.1179.

64. Jiang, L., and Zuo, X.-N. (2016). Regional homogeneity: a multimodal, multiscale neuroimaging marker of the human connectome. Neuroscientist *22*, 486–505. https://doi.org/10.1177/1073858415595004.

65. Zou, Q.-H., Zhu, C.-Z., Yang, Y., Zuo, X.-N., Long, X.-Y., Cao, Q.-J., Wang, Y.-F., and Zang, Y.-F. (2008). An improved approach to detection of amplitude of low-frequency fluctuation (ALFF) for resting-state fMRI: fractional ALFF. J. Neurosci. Methods *172*, 137–141. https://doi.org/10.1016/j.jneumeth.2008.04.012.

66. Cherubini, A., Caligiuri, M.E., Péran, P., Sabatini, U., Cosentino, C., and Amato, F. (2016). Importance of multimodal MRI in characterizing brain tissue and its potential application for individual age prediction. IEEE J. Biomed. Health Inform. *20*, 1232–1239. https://doi.org/10.1109/JBHI.2016.2559938.

67. Salih, A., Boscolo Galazzo, I., Raisi-Estabragh, Z., Rauseo, E., Gkontra, P., Petersen, S.E., Lekadir, K., Altmann, A., Radeva, P., and Menegaz, G. (2021). Brain age estimation at tract group level and its association with daily life measures, cardiac risk factors and genetic variants. Sci. Rep. *11*, 20563. https://doi.org/10.1038/s41598-021-99153-8.

68. Hahn, T., Fisch, L., Ernsting, J., Winter, N.R., Leenings, R., Sarink, K., Emden, D., Kircher, T., Berger, K., and Dannlowski, U. (2021). From 'loose fitting' to high-performance, uncertainty-aware brain-age modelling. Brain *144*, e31. https://doi.org/10.1093/brain/awaa454.

69. DeLisi, L.E., Sakuma, M., Tew, W., Kushner, M., Hoff, A.L., and Grimson, R. (1997). Schizophrenia as a chronic active brain process: a study of progressive brain structural change subsequent to the onset of schizophrenia. Psychiatry Res. *74*, 129–140.

70. Magalingam, K.B., Radhakrishnan, A., Ping, N.S., and Haleagrahara, N. (2018). Current concepts of neurodegenerative mechanisms in Alzheimer's disease. BioMed Res. Int. *2018*, 3740461.

71. White, K.E., and Cummings, J.L. (1996). Schizophrenia and Alzheimer's disease: clinical and pathophysiologic analogies. Compr. Psychiatr. *37*, 188–195. https://doi.org/10.1016/S0010-440X(96)90035-8.

72. Jack, C.R., Vemuri, P., Wiste, H.J., Weigand, S.D., Aisen, P.S., Trojanowski, J.Q., Shaw, L.M., Bernstein, M.A., Petersen, R.C., Weiner, M.W., et al. (2011). Evidence for ordering of Alzheimer disease biomarkers. Arch. Neurol. *68*, 1526–1535.

73. Rodrigues-Amorim, D., Rivera-Baltanás, T., López, M., Spuch, C., Olivares, J.M., and Agís-Balboa, R.C. (2017). Schizophrenia: a review of potential biomarkers. J. Psychiatr. Res. *93*, 37–49. https://doi.org/10.1016/j.jpsychires.2017.05.009.

74. Denissen, S., Engemann, D.A., De Cock, A., Costers, L., Baijot, J., Laton, J., Penner, I.-K., Grothe, M., Kirsch, M., D'hooghe, M.B., et al. (2022). Brain age as a surrogate marker for cognitive performance in multiple sclerosis. Eur. J. Neurol. *29*, 3039–3049. https://doi.org/10.1111/ene.15473.

75. Schnack, H.G., van Haren, N.E.M., Nieuwenhuis, M., Hulshoff Pol, H.E., Cahn, W., and Kahn, R.S. (2016). Accelerated brain aging in schizophrenia: a longitudinal pattern recognition study. Am. J. Psychiatr. *173*, 607–616. https://doi.org/10.1176/appi.ajp.2015.15070922.

76. Hofmann, S.M., Beyer, F., Lapuschkin, S., Goltermann, O., Loeffler, M., Müller, K.R., Villringer, A., Samek, W., and Witte, A.V. (2022). Towards the interpretability of deep learning models for multi-modal neuroimaging: finding structural changes of the ageing brain. Neuroimage *261*, 119504. https://doi.org/10.1016/j.neuroimage.2022.119504.

77. O'Shea, A., Cohen, R.A., Porges, E.C., Nissim, N.R., and Woods, A.J. (2016). Cognitive aging and the hippocampus in older adults. Front. Aging Neurosci. *8*, 298.

78. Jenness, J.L., Peverill, M., Miller, A.B., Heleniak, C., Robertson, M.M., Sambrook, K.A., Sheridan, M.A., and McLaughlin, K.A. (2021). Alterations in neural circuits underlying emotion regulation following child

maltreatment: a mechanism underlying trauma-related psychopathology. Psychol. Med. *51*, 1880–1889. https://doi.org/10.1017/S0033291720000641.

79. Truelove-Hill, M., Erus, G., Bashyam, V., Varol, E., Sako, C., Gur, R.C., Gur, R.E., Koutsouleris, N., Zhuo, C., Fan, Y., et al. (2020). A multidimensional neural maturation index reveals reproducible developmental patterns in children and adolescents. J. Neurosci. *40*, 1265–1275. https://doi.org/10.1523/JNEUROSCI.2092-19.2019.

80. von Hippel, P.T. (2015). The heterogeneity statistic I2 can be biased in small meta-analyses. BMC Med. Res. Methodol. *15*, 35.

81. Belkin, M., Hsu, D., Ma, S., and Mandal, S. (2019). Reconciling modern machine-learning practice and the classical bias–variance trade-off. Proc. Natl. Acad. Sci. USA *116*, 15849–15854. https://doi.org/10.1073/pnas.1903070116.

82. Li, Y., Li, F., Wang, P., Zhu, X., Liu, S., Qiu, M., Zhang, J., and Zeng, X. (2016). Estimating the brain pathological age of Alzheimer's disease patients from MR image data based on the separability distance criterion. Phys. Med. Biol. *61*, 7162–7186. https://doi.org/10.1088/0031-9155/61/19/7162.

83. Clausen, A.N., Fercho, K.A., Monsour, M., Disner, S., Salminen, L., Haswell, C.C., Rubright, E.C., Watts, A.A., Buckley, M.N., Maron-Katz, A., et al. (2022). Assessment of brain age in posttraumatic stress disorder: findings from the ENIGMA PTSD and brain age working groups. Brain Behav. *12*, e2413. https://doi.org/10.1002/brb3.2413.

84. Han, L.K.M., Dinga, R., Leenings, R., Hahn, T., Cole, J., Aftanas, L., Amod, A., Besteher, B., Colle, R., Corruble, E., et al. (2022). A Large-Scale ENIGMA Multisite Replication Study of Brain Age in Depression. https://doi.org/10.1101/2022.08.29.505635.

85. Han, L.K.M., Dinga, R., Hahn, T., Ching, C.R.K., Eyler, L.T., Aftanas, L., Aghajani, M., Aleman, A., Baune, B.T., Berger, K., et al. (2021). Brain aging in major depressive disorder: results from the ENIGMA major depressive disorder working group. Mol. Psychiatr. *26*, 5124–5139. https://doi.org/10.1038/s41380-020-0754-0.

86. West, D.B. (2001). Introduction to Graph Theory (Prentice hall Upper Saddle River).

87. Nickerson, L.D., Smith, S.M., Öngür, D., and Beckmann, C.F. (2017). Using dual regression to investigate network shape and amplitude in functional connectivity analyses. Front. Neurosci. *11*, 115. https://doi.org/10.3389/fnins.2017.00115.

Patterns, Volume 4

## Supplemental information

# A systematic review of multimodal brain age studies: Uncovering a divergence between model accuracy and utility

**Robert J. Jirsaraie, Aaron J. Gorelik, Martins M. Gatavins, Denis A. Engemann, Ryan Bogdan, Deanna M. Barch, and Aristeidis Sotiras**

**SUPPLEMENTAL MATERIAL**

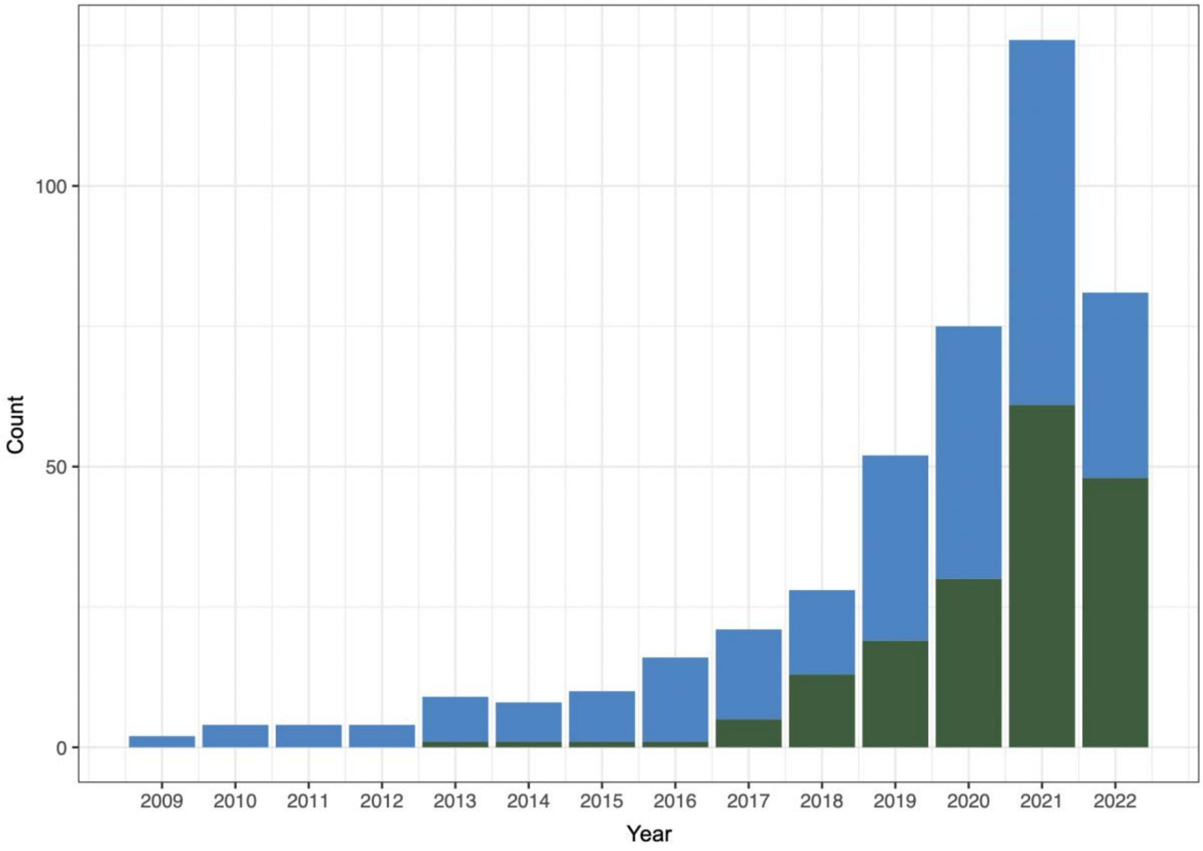


**Figure S1. The proportion of brain age articles that contain the word "multimodal" increases over time.** This histogram shows the total number of articles published in a given year that used the term "brain age" in their title, abstract, or keywords. The green shaded bars are the portion of brain age studies that either mentioned or referenced the term "multimodal" anywhere throughout the article. The blue shaded bars represent the brain age articles that did not use the term multimodal.

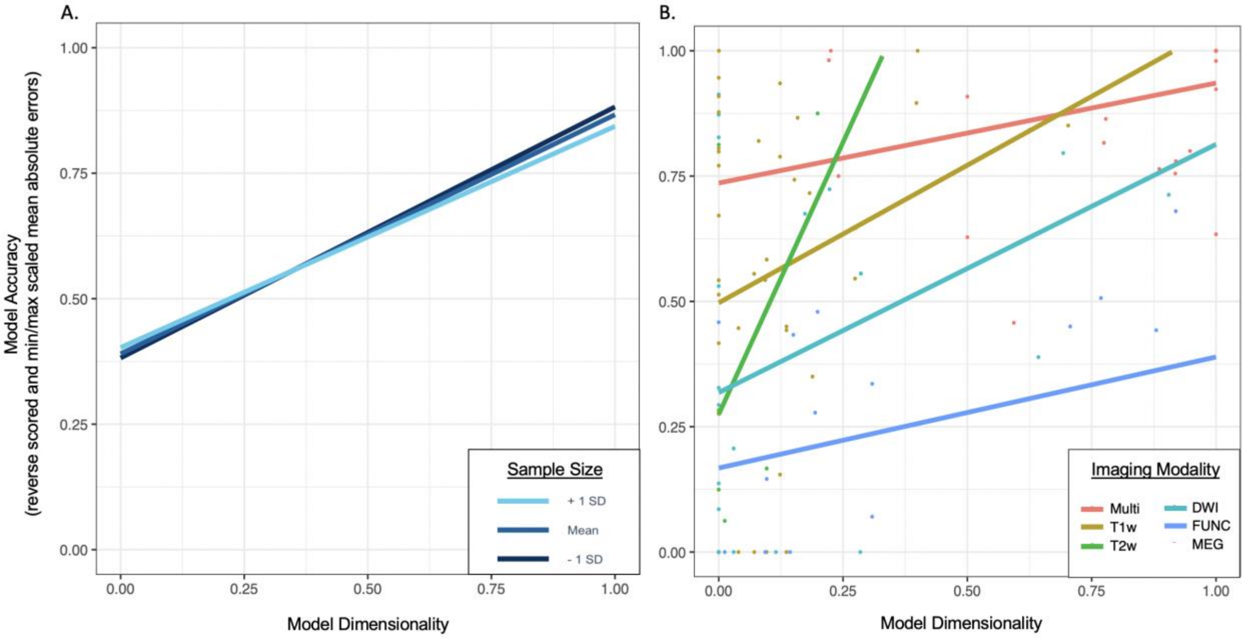


**Figure S2. The relationship between model accuracy and number of features was not moderated by sample size or imaging modality. A.** Brain age models with a greater number of neuroimaging features tend to have better accuracy regardless of the sample size used to train and evaluate them ($\beta$ = -0.05, p = 0.71). **B.** When evaluating models within a given imaging modality, model accuracy was consistently correlated with the number of features used. Scaled MAEs with a score of 1 were the most accurate within a given study while those with a 0 were the least accurate.

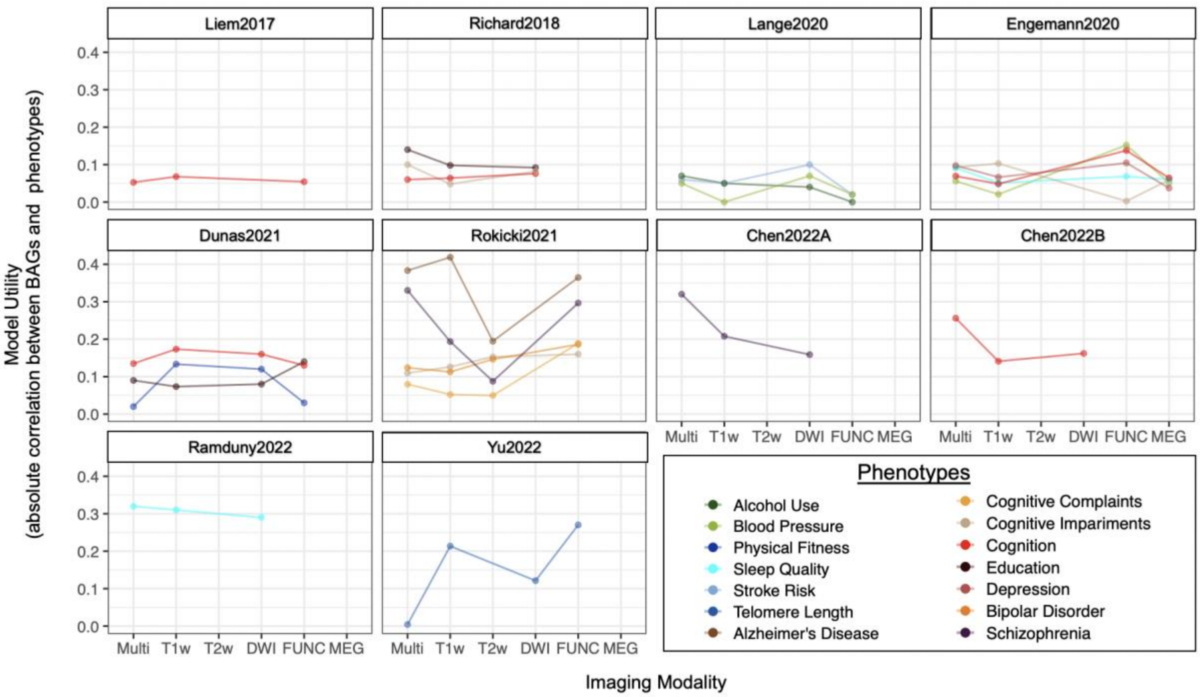


**Figure S3. The utility of brain age models varied considerably by the phenotype of interest.** When evaluating studies that analyzed multiple phenotypes it became apparent that the utility of brain age models was largely dependent on the phenotype in question. Each colored line represents the utility of brain age models for a specific phenotype.

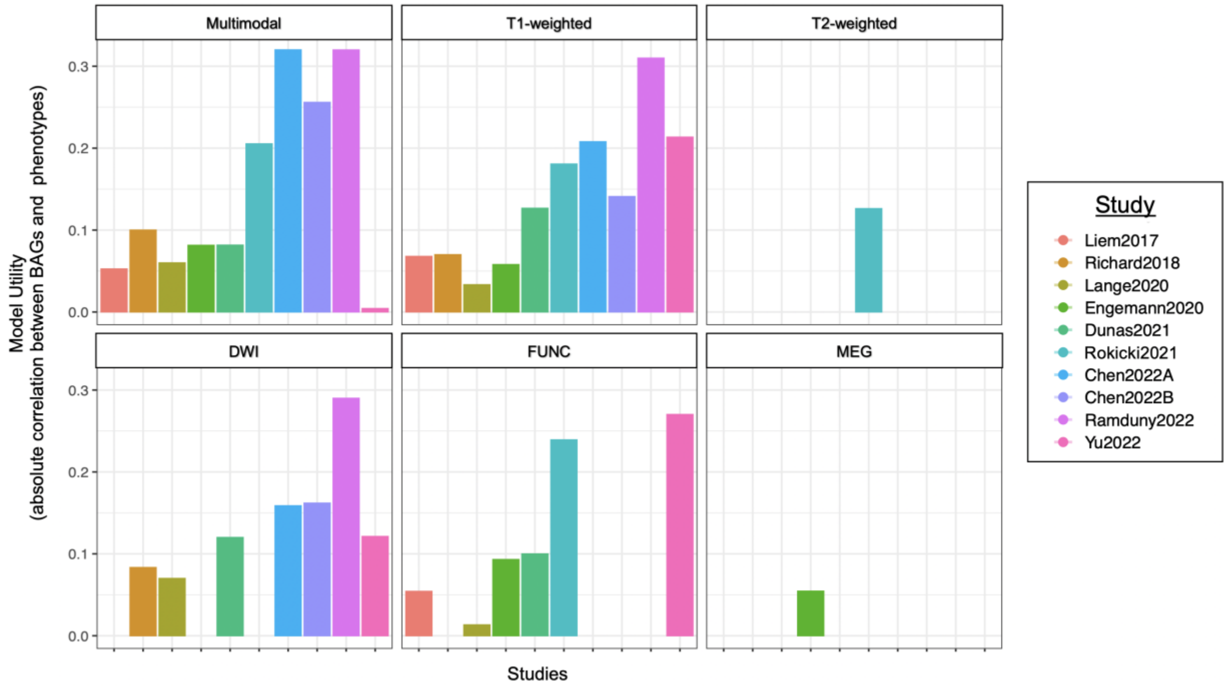


**Figure S4. The utility of brain age models varied considerably between imaging modalities and across studies.** Each bar denotes that the utility of brain age models that were derived from a given imaging source for a specific study. There was considerably heterogeneity between in model utility across the ten studies included in this review.

**Table S1.** Neuroimaging Modalities and Feature Types.

| Study | Structural MRI | Functional MRI | Diffusion MRI | Miscellaneous |
|---|---|---|---|---|
| Erus et al. (2015)[maps] | CT, WM, VN | - | FA, ADC | - |
| Liem et al. (2017) | CT, SA, subcortical volume | rsFC (197, 444) | - | - |
| Richard et al. (2018) | CT, SA, CVol | - | FA, MD, RD, AD | - |
| Cole (2020) | CT, SA, CVol | rsFC, taskFC | FA, MD, MO, L1:3, ICVF, OD, ISOVF | T2-FLAIR, T2* |
| de Lange et al. (2020) | CT, SA, CVol | rsFC (ICA, PCorr) | FA, MD, AD, RD, MO | global WMH volume |
| Engemann et al. (2020) | CT, SA, subcortVol | rsFC | - | MEG |
| Galdi et al. (2020) | Regional volumes | - | FA, MD, AD, RD, MK, ICVF, ISOVF, OD, OD1, OD2 | T1w/T2w ratio, constructed MSN |
| Hu et al. (2020) | LGI, MC, AC, CT, SA, CVol | rsFC | - | - |

| | | | | |
|---|---|---|---|---|
| Niu et al. (2020) | CVol | ALFF, ReHo | FA, MD | - |
| Zhang et al. (2020) | CT, SA, CVol | - | connectome | - |
| Dadi et al. (2021) | CVol, subcortical volume | rsFC (ICA) | FA, MD, MO, ICVF, ISOVF, OD | - |
| Dunas et al. (2021)[maps] | CVol maps, WMVol, CSF maps | rsFC | FA intensity map | - |
| Luna et al. (2021) | CT, CVol, SA, MC | - | connectome | - |
| Rokicki et al. (2021) | CT, SA, CVol | - | - | T1w/T2w signal ratio, CBF |
| Xifra-Porxas et al. (2021)[maps] | GM, WM, CSF | - | - | MEG maps |
| Chen et al. (2022a) | CVol, CT | - | FA, MD | - |
| Chen et al. (2022b) | CVol, CT | - | FA, MD | - |
| Huang et al. (2022) | CVol, WM Vol | ALFF, ReHo, rsFC DC | FA, MD, RD, AD | - |
| Ramduny et al. (2022) | CVol, subcortical volume | - | FA, MD | - |

| | | | | |
|---|---|---|---|---|
| Yu et al. (2022) | CT, subcortical volume | rsFC | connectome | - |

*Note.* [maps] – maps used to denote that spatial maps were the input features used for a given predicative algorithm.

**Structural (T1w) MRI measures:** CVol (cortical gray matter volume), WMVol (white matter volume), VNVol (ventricle volume), CT (cortical thickness), SA (cortical surface area), LGI (local gyrification index), MC (mean curvature), AC (average convexity), CSF (cerebrospinal fluid intensity)

**Functional MRI measures:** rsFC (resting-state functional connectivity): ICA (defining nodes using independent component analysis), PCorr (using partial correlations between time series at different parcellated regions); ALFF (amplitude of low-frequency fluctuations), ReHo (regional homogeneity), DC (degree centrality in a functional connectivity network), taskFC (task-based MRI functional connectivity)

**Diffusion weighted imaging measures:** FA (fractional anisotropy), MD (mean diffusivity), RD (radial diffusivity), AD (axial diffusivity), ADC (apparent coefficient of diffusion), ICVF (intracellular volume fraction), ISOVF (isotropic volume fraction), MO (mode of anisotropy), MK (mean kurtosis), OD (total orientation dispersion), OD1 & OD2 (orientation dispersion along primary (1) and secondary (2) axes), L1-3 (diffusion tensor along primary, secondary, and tertiary axes of an ellipse), Connectome (tractography-derived structural connectivity matrix).

**Other measures:** CBF (cerebral blood flow), MSN (morphometric similarity networks), WMH (white matter hyperintensities)

**Table S2.** Accuracy of brain age models from all twenty studies included in this review.

| Study | Multimodal (MAE \| r \| COD) | T1-weighted MRI (MAE \| r \| COD) | T2-weighted MRI (MAE \| r \| COD) | Diffusion MRI (MAE \| r \| COD) | Functional MRI (MAE \| r \| COD) | MEG (MAE \| r \| COD) |
|---|---|---|---|---|---|---|
| Erus et al. (2015)$_{maps}$ | 1.00 \| 0.88 \| 0.79 | 0.49 \| 0.69 \| 0.47 | - | 0.98 \| 0.84 \| 0.71 | - | - |
| Liem et al. (2017) | 1.00 \| NA \| NA | 0.39 \| NA \| NA | - | 0.54 \| NA \| NA | - | - |
| Richard et al. (2018) | 1.00 \| 0.85 \| 0.73 | 0.46 \| 0.73 \| 0.53 | - | 0.40 \| 0.71 \| 0.50 | - | - |
| Cole (2020) | 1.00 \| 0.78 \| 0.62 | 0.74 \| 0.69 \| 0.47 | 0.09 \| 0.32 \| 0.10 | 0.84 \| 0.73 \| 0.53 | 0.14 \| 0.33 \| 0.11 | - |
| de Lange et al. (2020) | 1.00 \| 0.54 \| 0.30 | 0.72 \| 0.47 \| 0.22 | - | 0.83 \| 0.49 \| 0.24 | 0.00 \| 0.00 \| 0.00 | - |
| Engemann et al. (2020) | 0.85 \| NA \| NA | 0.35 \| NA \| NA | - | - | 0.45 \| NA \| NA | 0.00 \| NA \| NA |
| Galdi et al. (2020) | 1.00 \| 0.78 \| 0.61 | 0.21 \| 0.47 \| 0.22 | - | 0.50 \| 0.58 \| 0.34 | - | - |
| Hu et al. (2020) | 1.00 \| NA \| NA | 0.91 \| NA \| NA | - | - | 0.00 \| NA \| NA | - |
| Niu et al. (2020) | 0.91 \| 0.87 \| 0.75 | 0.54 \| 0.82 \| 0.67 | - | 0.10 \| 0.76 \| 0.58 | 0.20 \| 0.77 \| 0.60 | - |
| Zhang et al. (2020) | 1.00 \| NA \| NA | 0.53 \| NA \| NA | - | 0.00 \| NA \| NA | - | - |
| Dadi et al. (2021) | 0.89 \| NA \| NA | 0.88 \| NA \| NA | - | 0.80 \| NA \| NA | 0.00 \| NA \| NA | - |
| Dunas et al. (2021)$_{maps}$ | 0.88 \| NA \| NA | 0.62 \| NA \| NA | - | 0.67 \| NA \| NA | 0.00 \| NA \| NA | - |
| Luna et al. (2021) | 1.00 \| NA \| NA | 0.00 \| NA \| NA | - | 0.71 \| NA \| NA | - | - |

| | | | | | | |
|---|---|---|---|---|---|---|
| Rokicki et al. (2021) | 1.00 \| 0.87 \| 0.77 | 0.56 \| 0.76 \| 0.58 | 0.62 \| 0.78 \| 0.61 | - | 0.36 \| 0.69 \| 0.47 | - |
| Xifra-Porxas et al. (2021)$_{maps}$ | 1.00 \| NA \| NA | 0.87 \| NA \| NA | - | - | - | 0.00 \| NA \| NA |
| Chen et al. (2022a) | 1.00 \| 0.97 \| 0.94 | 0.54 \| 0.96 \| 0.94 | - | 0.00 \| 0.94 \| 0.88 | - | - |
| Chen et al. (2022b) | 1.00 \| 0.89 \| 0.80 | 0.65 \| 0.87 \| 0.76 | | 0.00 \| 0.76 \| 0.58 | - | - |
| Huang et al. (2022) | 0.75 \| 0.84 \| 0.70 | 0.54 \| 0.79 \| 0.62 | - | 0.08 \| 0.72 \| 0.52 | 0.00 \| 0.70 \| 0.49 | - |
| Ramduny et al. (2022) | 0.92 \| 0.74 \| 0.54 | 1.00 \| 0.76 \| 0.58 | - | 0.00 \| 0.64 \| 0.41 | - | - |
| Yu et al. (2022) | 0.63 \| 0.54 \| 0.29 | 0.97 \| 0.55 \| 0.30 | - | 0.00 \| 0.52 \| 0.27 | 0.44 \| 0.46 \| 0.21 | - |

*Note.* Evaluation metrics regarding the accuracy of brain age models are reported. If a given study evaluated multiple models derived from a single imaging modality then the metrics across all model variants were averaged. To quantify mean absolute error, we first min/max normalized the prediction errors from within a given study, and subsequently, inverted them. A score of 1 suggests that models from these feature types were the most accurate within a given study, whereas a score of 0 denotes that the least accurate models within a given study. Therefore, this definition of model accuracy most reflected relative performance within a given study as opposed to differences between studies. The second and third accuracy metrics reported in each table cell is the correlation and coefficient of determination, respectively. Such accuracy metrics were recorded if the original study reported the Pearson correlation between chronological and brain ages.

**Table S3.** Utility of brain age models from a subset of ten studies included in this review.

| Study | Multimodal | T1-weighted MRI | T2-weighted MRI | Diffusion MRI | Functional MRI | MEG |
|---|---|---|---|---|---|---|
| Liem et al. (2017) | 0.05 | 0.06 | - | 0.05 | - | - |
| Richard et al. (2018) | 0.10 | 0.07 | - | 0.08 | - | - |
| de Lange et al. (2020) | 0.06 | 0.03 | - | 0.07 | 0.01 | - |
| Engemann et al. (2020) | 0.08 | 0.06 | - | - | 0.09 | 0.05 |
| Dunas et al. (2021)$_{maps}$ | 0.08 | 0.13 | - | 0.12 | 0.10 | - |
| Rokicki et al. (2021) | 0.21 | 0.18 | 0.13 | - | 0.23 | - |
| Chen et al. (2022a) | 0.32 | 0.21 | - | 0.16 | - | - |
| Chen et al. (2022b) | 0.26 | 0.14 | | 0.16 | - | - |
| Ramduny et al. (2022) | 0.32 | 0.31 | - | 0.29 | - | - |
| Yu et al. (2022) | 0.01 | 0.21 | - | 0.12 | 0.27 | - |

*Note.* Evaluation metrics regarding the utility of brain age models are reported. If a given study evaluated multiple models derived from a single imaging modality then the metrics across all model variants were averaged. Model utility was quantified as the absolute value of the correlation between brain age gaps and phenotypes of interest. Taking the absolute value was preferred to more broadly reflect the strengthen of the effect size across multiple phenotypes.
